# Stress response requires an efficient connection between glycogen and central carbon metabolism by phosphoglucomutases in cyanobacteria

**DOI:** 10.1093/jxb/erac474

**Published:** 2022-12-01

**Authors:** Pablo Ortega-Martínez, Miguel Roldán, Sandra Díaz-Troya, Francisco J Florencio

**Affiliations:** Instituto de Bioquímica Vegetal y Fotosíntesis, Universidad de Sevilla, Consejo Superior de Investigaciones Científicas, Américo Vespucio 49, Sevilla, 41092, Spain; Departamento de Bioquímica Vegetal y Biología Molecular, Facultad de Biología, Universidad de Sevilla, Profesor García González s/n, Sevilla, 41012, Spain; Departamento de Bioquímica Vegetal y Biología Molecular, Facultad de Biología, Universidad de Sevilla, Profesor García González s/n, Sevilla, 41012, Spain; Instituto de Bioquímica Vegetal y Fotosíntesis, Universidad de Sevilla, Consejo Superior de Investigaciones Científicas, Américo Vespucio 49, Sevilla, 41092, Spain; Departamento de Bioquímica Vegetal y Biología Molecular, Facultad de Biología, Universidad de Sevilla, Profesor García González s/n, Sevilla, 41012, Spain; Instituto de Bioquímica Vegetal y Fotosíntesis, Universidad de Sevilla, Consejo Superior de Investigaciones Científicas, Américo Vespucio 49, Sevilla, 41092, Spain; Departamento de Bioquímica Vegetal y Biología Molecular, Facultad de Biología, Universidad de Sevilla, Profesor García González s/n, Sevilla, 41012, Spain; INRAE-Bordeaux, France

**Keywords:** Cyanobacteria, environmental stress, glycogen, high light, nitrogen metabolism, phosphoglucomutase, phosphohexomutase

## Abstract

Glycogen and starch are the main storage polysaccharides, acting as a source of carbon and energy when necessary. Interconversion of glucose-1-phosphate and glucose-6-phosphate by phosphoglucomutases connects the metabolism of these polysaccharides with central carbon metabolism. However, knowledge about how this connection affects the ability of cells to cope with environmental stresses is still scarce. The cyanobacterium *Synechocystis* sp. PCC 6803 has two enzymes with phosphoglucomutase activity, PGM (phosphoglucomutase) and PMM/PGM (phosphomannomutase/phosphoglucomutase). In this work, we generated a null mutant of PGM (∆PGM) that exhibits very reduced phosphoglucomutase activity (1% of wild type activity). Although this mutant accumulates moderate amounts of glycogen, its phenotype resembles that of glycogen-less mutants, including high light sensitivity and altered response to nitrogen deprivation. Using an on/off arsenite promoter, we demonstrate that PMM/PGM is essential for growth and responsible for the remaining phosphoglucomutase activity in the ∆PGM strain. Furthermore, overexpression of PMM/PGM in the ∆PGM strain is enough to revoke the phenotype of this mutant. These results emphasize the importance of an adequate flux between glycogen and central carbon metabolism to maintain cellular fitness and indicate that although PGM is the main phosphoglucomutase activity, the phosphoglucomutase activity of PMM/PGM can substitute it when expressed in sufficient amounts.

## Teaser

*Synechocystis* has two enzymes with phosphoglucomutase activity: PGM and PMM/PGM. PGM is responsible for most of the phosphoglucomutase activity, but PMM/PGM is sufficient to sustain glycogen metabolism and its overexpression overrides the stress sensitivity phenotype of a PGM-null mutant.

## Introduction

Cyanobacteria are widespread photosynthetic microorganisms of enormous ecological, evolutionary, and biotechnological importance. They play an essential role in supporting the trophic chains through their oxyphototrophic metabolism, mainly in aquatic ecosystems, and are of great importance in carbon and nitrogen cycles. During recent years, these phototrophs have emerged as potential tools for biotechnological purposes, although the side effects of modifying their metabolic carbon flux are a drawback that requires further exploration (Luan *et al.*, 2019).

**Fig. 1.**
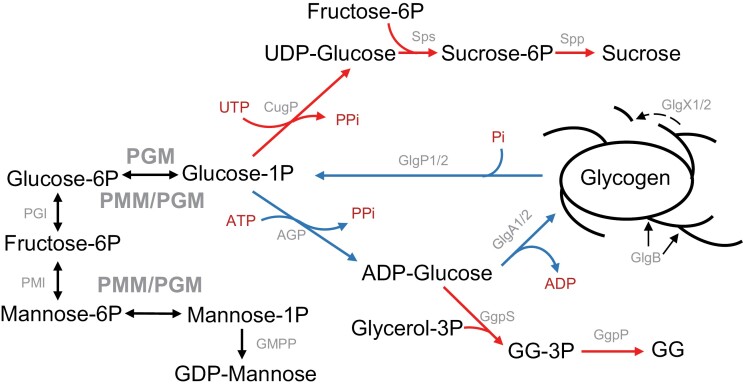
Schematic representation of the connection between central carbon metabolism and the pathways for glycogen and glucosylglycerol synthesis in *Synechocystis*. AGP, ADP-glucose pyrophosphorylase; CugP, UTP-glucose-1-phosphate uridylyltransferase; GgpP, glucosylglycerolphosphate phosphatase; GgpS, glucosylglycerolphosphate synthase; GlgA1/2, glycogen synthases; GlgB, glycogen branching enzyme; GlgP1/2, glycogen phosphorylases; GlgX1/2, glycogen debranching enzyme; GG, glucosylglycerol; GG-3P, glucosylglycerol-3-phosphate; GMPP, GDP-mannose pyrophosphorylase; PGI, glucose-6-phosphate isomerase; PGM, phosphoglucomutase; PMI, mannose-6-phosphate isomerase; PMM/PGM, phosphomannomutase/phosphoglucomutase; Spp, sucrose-phosphate phosphatase; Sps, sucrose-phosphate synthase. Blue arrows, reactions for glycogen synthesis and degradation; red arrows, reactions for synthesis of osmolites glucosylglycerol and sucrose; black arrows, reactions connecting glucose-1P and mannose-1P with central carbon metabolism.

Photosynthesis supplies the energy and metabolites required to support the vast array of biosynthetic processes, development, and cell division. The ability to store part of the photosynthates as polysaccharide gives the cells a remarkable plasticity: its accumulation and mobilization, in addition to providing cells with carbon on demand, act as a buffer that helps maintain energy homeostasis and allows cells to cope with a changing environment (Cano *et al.*, 2018). Glycogen is the main polysaccharide in cyanobacteria, but can be found in both prokaryotic (including archeobacteria) and eukaryotic cells (animal and fungi) (Ball and Morell, 2003; Cifuente *et al.*, 2019). In cyanobacteria, glycogen plays an essential role to face some environmental stresses such as diel cycles, high irradiance, or nutrient-limited environments, especially nitrogen deprivation in non-diazotrophic cyanobacteria (Cano *et al.*, 2018; Forchhammer and Schwarz, 2019; Welkie *et al.*, 2019). Glycogen synthesis starts with the formation of ADP-glucose (ADPGlc) from glucose-1-phosphate (G1P) and ATP by ADP-glucose pyrophosphorylase (AGP). Glycogen synthase (GlgA) employs ADP-glucose to elongate the growing polysaccharide chain. Finally, the branching enzyme (GlgB) introduces α-1,6 ramifications. For glycogen catabolism, glucose chains are shortened from their non-reducing ends by glycogen phosphorylases (GlgPs), liberating G1P. Once a branching point is reached, the α-1,6 glucosidic ramifications are removed by the hydrolase activity of the debranching enzyme (GlgX) (Fig. 1). The concerted action of these enzymes provides the characteristic architecture of glycogen granules (Ball *et al.*, 2015; Cifuente *et al.*, 2019).

Since glycogen metabolism begins and ends with G1P, the flux between glycogen and central carbon metabolism relies on the reversible conversion of G1P and G6P by enzymes with phosphoglucomutase activity. These proteins belong to the phosphohexomutase superfamily. Phosphohexomutases are present in all kingdoms of life, playing a critical role in a vast array of metabolic processes. These proteins share a common catalytic mechanism and similar structural domains despite using different phosphosugar substrates such as glucose, mannose, glucosamine, or *N*-acetylglucosamine (Shackelford *et al.*, 2004). In fact, the preferred substrate employed by these enzymes has traditionally been used to classify them into subgroups: phosphoglucomutase (PGM), phosphomannomutase/phosphoglucomutase (PMM/PGM), phosphoglucosamine mutase (PNGM), phoshoacetylglucosamine mutase (PAGM), phosphopentomutase, and glucose-1,6-biphosphate synthase (Stiers *et al.*, 2017). These enzymes are phosphorylated on a conserved serine residue and their catalytic cycle occurs by phosphoryl exchanges between the enzyme and the substrate, allowing the transfer of the phosphate of the phosphosugar substrate from C6 to C1 or vice versa. Briefly, the process begins with the transfer of the phosphate from the catalytic phosphoserine to the substrate, generating a bisphosphorylated intermediate product. This dephospho-form of the enzyme acquires a more flexible conformation, allowing the intermediate product to reorient in a 180° flip. In that state, the serine residue reacquires the phosphate and a sugar monophosphate is released (Regni *et al.*, 2002, 2004; Stiers *et al.*, 2019). These enzymes need magnesium as a cofactor and present substrate inhibition by catalytic cleft occupancy of G1P in the dephospho-form of the enzyme, an effect alleviated by glucose-1,6-bisphosphate (G1,6BP) as an activator of autophosphorylation (Ray and Roscelli, 1964; Naught and Tipton, 2005).

*Synechocystis* sp. PCC 6803 (hereafter *Synechocystis*) is a cyanobacterium that exclusively accumulates glycogen as storage polysaccharide. The genome of *Synechocystis* presents three genes coding for enzymes belonging to the phosphohexomutase superfamily: *sll1758* coding for a predicted protein of the PNGM subgroup; and *sll0726* and *slr1334*, coding for PGM and PMM/PGM, respectively, both enzymes with phosphoglucomutase activity (Liu *et al.*, 2013). Recent findings in *Synechocystis* have highlighted the role of PGM as a key regulatory element in glycogen metabolism (Doello *et al.*, 2022). When nitrogen starved, *Synechocystis* enters a dormant-like state (Neumann *et al.*, 2021). Proteomic studies showed that PGM is highly phosphorylated at Ser47 (in the regulatory latch domain, a distinct serine from that in the catalytic cleft) under nitrogen deprivation (Spät *et al.*, 2018). This phosphorylation maintains PGM in an inactive state and is essential for survival in these conditions (Doello *et al.*, 2022). PMM/PGM has recently been described to have reversible *in vitro* activity as a G1,6BP synthase, using both fructose-1,6-biphosphate and either G1P or G6P. This makes PMM/PGM a potential key regulator of the enzymes from the phosphohexomutase superfamily (Neumann *et al.*, 2022).

As previously indicated, phosphoglucomutase activity is a key point in the connection between glycogen and central carbon metabolism. However, knowledge about the effects of limited G1P–G6P interconversion on cell fitness and the role of PGM and PMM/PGM in this process is scarce. Here, we generated and characterized mutant strains of the two enzymes with phosphoglucomutase activity in *Synechocystis*. We show that PGM is required for an adequate connection between glycogen and central carbon metabolism, and its absence causes a reduced fitness to stresses such as high light or nitrogen deprivation. However, the response to these stresses is recovered when PMM/PGM is overexpressed, indicating a partial metabolic redundancy of these enzymes. All of this emphasizes the importance of an optimal management of carbon reserves in cyanobacteria.

## Materials and methods

### Strain and culture conditions

*Synechocystis* sp. PCC 6803 strains were cultured in BG11 medium (Rippka *et al.*, 1979) supplemented with NaHCO_3_ to a final concentration of 12 mM (BG11C) except for carbon-limited experiments where 5 mM or no NaHCO_3_ was used. Cultures were grown at 30 °C under continuous light (4500k LED lights, 50 µE m^−2^ s^−1^) in non-bubbled Erlenmeyer flasks at 100 rpm or in conical flasks bubbled with a stream of 1% (v/v) CO_2_ in air. For nitrogen deficiency experiments, cultures were washed twice by centrifugation (10 min at 7000 *g*) and resuspension in nitrogen-free medium (BG11_0_C), and finally resuspended at 1 OD_750 nm_ in BG11_0_C. For inducer (NaAsO_2_) removal experiments, cultures were washed twice by centrifugation (10 min at 7000 *g*) and resuspension in media without inducer, and finally resuspended at 1 OD_750 nm_. Experiments under light/dark conditions were performed in Erlenmeyer flasks on a New Brunswick™ Innova® 43/43R shaker at 40 µE m^−2^ s^−1^.When required, media were supplemented with Bacto agar (Difco) [1% (w/v)] or the required antibiotics (50 µg ml^−1^ kanamycin, 20 µg ml^−1^ chloramphenicol, 2.5 µg ml^−1^ spectinomycin, 2.5 µg ml^−1^ streptomycin, 50 µg ml^−1^ nourseothricin, and 5 µg ml^−1^ erythromycin).

### Generation of mutant strains

The ∆PGM strain lacking the *sll0726* gene was obtained by transformation of the wild-type (WT) strain with plasmid pGT∆sll0726::Km, which allows for the deletion of the complete *sll0726* ORF. To generate this plasmid, regions upstream and downstream of *sll0726* were amplified with primers OL68/OL69 and OL70/OL71, respectively, which introduce a *Bam*HI restriction site, and joined by overlapping PCR with primers OL68/OL71. This fragment was cloned into pGEM-T and a kanamycin resistance cassette was introduced in the generated *Bam*HI site. The ∆AGP∆PGM strain was obtained by transformation of the ∆AGP strain with plasmid pGT∆sll0726::Km.

Plasmid pGT∆slr1334::Ery was used to generate the ∆PMM* strain, a partially segregated ∆*slr1334* mutant. This plasmid was also constructed with the insertion of a DNA fragment with a *Bam*HI site between the upstream (primers OL72/OL73b) and downstream (primers OL74b/OL75) regions of *slr1334*, obtained by overlapping PCR with primers OL72 and OL75. This fragment was cloned into pGEM-T and an erythromycin resistance cassette was introduced in *Bam*HI. This construction does not delete the complete *slr1334* ORF, as it partially overlaps with the hypothetical protein encoded by *sll1219*.

To generate the PMM/PGM-overexpressing strains OE:M and ∆PGM_OE:M, cultures of the WT and ∆PGM strains, respectively, were transformed with plasmid pnrsD_PcpcB_slr1334_SpR. To obtain this plasmid, the *slr1334* ORF was extracted from plasmid pET28_slr1334 (plasmid described in the next section) and cloned in *Xba*I/*Xho*I in plasmid pnrsD_PcpcB_SpR.

The ∆PMM_P*ars*:M strain was obtained by introducing a regulated copy of *slr1334* in the WT background with plasmid pnrsD_arsB_slr1334_SpR (*slr1334* ORF from plasmid pET28_slr1334 cloned in *Xba*I/*Xho*I in plasmid pnrsD_ParsB_SpR) and then deleting endogenous *slr1334* with plasmid pGT∆slr1334::Ery in the presence of arsenite. For generation of the ∆PGM∆PMM_P*ars*:M strain, the ∆PMM_P*ars*:M strain was transformed with plasmid pGT∆sll0726::Km.

Strains and primers used in this work can be found in Table 1 and Supplementary Table S1, respectively. All plasmids were verified by Sanger sequencing, and complete segregation of the mutants was confirmed by PCR.

**Table 1.** Strains used in this work

Name	Genotype	Reference
WT	Wild-type *Synechocystis* sp. PCC 6803	
∆AGP	∆*agp*::C.C1	Díaz-Troya *et al.* (2014)
∆PGM	∆*pgm*::CK1	This work
∆PMM^*a*^	∆*pmm/pgm*::Ery	This work
OE:M	*nrsD*::P*cpcB*:*pmm/pgm*:SpΩ	This work
∆PGM_OE:M	∆*pgm*::CK1, *nrsD*::P*cpcB*:*pmm/pgm*:SpΩ	This work
∆PMM_P*ars*:M	∆*pgm/pmm*::Ery, *nrsD*::P*arsB*:*pmm/pgm*:SpΩ	This work
∆PGM∆PMM_P*ars*:M	∆*pgm::*CK1,∆*pmm/pgm*::Ery, *nrsD*::P*arsB*:*pmm/pgm*:SpΩ	This work

### Purification of recombinant *Synechocystis* PGM and PMM/PGM, and generation of specific antibodies

The sequence of the complete ORFs encoding PGM and PMM/PGM were obtained from genomic *Synechocystis* DNA by PCR with primer pairs OL78/OL79 and OL80/OL81, respectively (sequences in Supplementary Table S1). Note that the fragment amplified for PGM did not include the sequence corresponding to the first 16 amino acids as depicted in Cyanobase, as they were not conserved in other cyanobacterial genomes. These sequences were cloned in *Nde*I/*Eco*RI in pET28, resulting in the plasmids pET28_sll0726 and pET28_slr1334. *Escherichia coli* transformation and recombinant protein expression were performed following the instructions of the manufacturer of the pET vectors (Novagen). In brief, *E. coli* BL21 (DE3) cells were transformed with these plasmids with a standard heat shock protocol and inoculated in kanamycin-supplemented Luria–Bertani medium. The expression of 6×His-tagged recombinant proteins was induced adding 0.2 mM isopropyl-β-d-thiogalactopyranoside (IPTG). After 24 h of incubation at 25 °C, cells were harvested by centrifugation and resuspended in 50 mM Tris–HCl pH 8.0, 500 mM NaCl, 1 mM phenylmethylsulfonyl fluoride (PMSF). Cells were lysed by sonication (20 kHz, 75 W) on ice for 3 min (in 30 s periods). Lysates were centrifuged at 20 000 *g* for 30 min and supernatants were filtered through 0.22 µM filters (Millipore). Recombinant PGM and PMM/PGM were purified by affinity chromatography with the Ni-NTA Purification System (NeoBiotech) following the manufacturer’s instructions. In brief, clarified supernatants were supplemented with imidazole to a final concentration of 25 mM and loaded onto Ni-NTA agarose columns. After washing with 50 mM Tris–HCl, pH 8.0, 500 mM NaCl, 25 mM imidazole, bound recombinant proteins were eluted with increasing concentrations of imidazole in 50 mM Tris–HCl pH 8.0, 500 mM NaCl. Next, purified proteins were desalted using PD-10 columns (GE Healthcare) pre-equilibrated with 50 mM Tris–HCl pH 8.0, 150 mM NaCl, 20% glycerol, and concentrated with 30K molecular weight cut-off Amicon Ultra centrifugal filter units (Millipore). Polyclonal antibodies against PGM and PMM/PGM recombinant proteins were generated in rabbits following standard immunization protocols in the Centro de Experimentación Animal Oscar Pintado (CITIUS).

### Oxygen evolution

A Clark-type oxygen electrode (Hansatech) was used to measure oxygen evolution in cultures in a 30 °C chamber during 10 min at 50 µmol photon m^−2^ s^−1^. To prevent carbon limitation, cultures were supplemented with 10 mM NaHCO_3_ just before measurements.

### Photosynthetic measurements

Chlorophyll fluorescence was measured by pulse amplitude modulation (PAM) fluorometry with a Dual-PAM-100 (Walz) using intact cells at room temperature. Before recording, cells were adapted in the dark for 10 min. A 250 ms saturation pulse (5000 µmol photons m^−2^ s^−1^) was set for determination of the effective quantum yield of PSII [Y(II)]. Y(II) was determined with the formula (*F*_m_ʹ–*F*_s_)/(*F*_m_ʹ) where *F*_m_ʹ is maximal fluorescence and *F*_s_ is basal fluorescence, both measured during exposure to actinic light at the same intensity used to culture the strains (50, 100, or 200 µE m^−2^ s^−1^). Other parameters analyzed were basal fluorescence in the dark (*F*_o_) and maximum fluorescence measured in the presence of 20 µM DCMU (*F*_m_).

### Glycogen content determination

Glycogen content was determined as described in Gründel *et al.* (2012) with minor modifications. A 2 ml aliquot of culture was harvested by centrifugation (15 000 *g* for 10 min at 4 °C) and stored at –20 °C after the supernatant was carefully removed. Frozen pellets were resuspended in 30% KOH, vigorously mixed by vortexing for 3 min, and incubated for 2 h at 95 °C. Cold ethanol was added for glycogen precipitation and incubated overnight at –20 °C. Glycogen was purified after two cycles of centrifugation (15 000 *g* for 10 min at 4 °C) and washed with cold ethanol, ensuring total elimination of KOH traces. After drying the pellet, it was resuspended in 100 mM sodium acetate (pH 5.2) and digested with 10 U of amyloglucosidase (form *Aspergillus niger*, Sigma) at 55 °C overnight in parallel with a calibration curve using glycogen (from bovine liver, Sigma). Glucose released in the process was quantified with a coupled enzyme reaction of glucose oxidase (from *A. niger*, Sigma) and peroxidase (from horseradish, Sigma) in the presence of *O*-dianisidine (Sigma) to a final concentration of 200 mg ml^–1^, 25 U ml^–1^, and 5 U ml^–1^, respectively, for 30 min at 30 °C. The reaction was stopped by adding H_2_SO_4_ to a final concentration of 4.8 N, and absorbance was registered at 540 nm on a Varioskan multiplate reader (Thermo Fisher Scientific).

### Determination of extracellular pyruvate and 2-oxoglutarate

Determination of metabolites in the extracellular medium was performed as in Díaz-Troya *et al.* (2020) with minor modifications. A 6 ml aliquot of culture samples was centrifuged (15 000 *g* for 10 min at 4 °C) and supernatants were quick-frozen in liquid nitrogen, lyophilized (VirTis BenchTop Pro Freeze dryer, SP Scientific), and stored at –20 °C until analyzed. Samples were resuspended in 600 µl of H_2_O, and 10–100 µl aliquots were analyzed by enzyme-coupled assays in 375 mM Tris–HCl (pH 7.5) with 0.11 mM NADH (and 50 mM NH_4_Cl for 2-oxoglutarate quantification). The reaction was triggered by the addition of 5 mU of lactate dehydrogenase (Sigma) or 12 mU of glutamate dehydrogenase (Sigma) for the determination of pyruvate or 2-oxoglutarate, respectively. When NADH oxidation was completed, the remaining NADH was measured spectrophotometrically at 340 nm to calculate the concentration of the metabolite compared with standard curves of known amounts of pyruvate (Sigma) and 2-oxoglutarate (Sigma).

### Quantification of intracellular osmolytes (GG and sucrose)

Frozen pellets from 4 ml culture aliquots were incubated in 1 ml of 80% ethanol for 4 h at 65 °C and the supernatants were recovered by centrifugation (15 000 *g* at room temperature for 5 min and dried in a vacuum centrifuge. Pellets were solubilized in 100 µl of ultrapure water and filtered through 0.20 µm filters (Millex-GN Nylon, Millipore). Samples (10 μl) were analyzed by HPLC in a Waters LC Module I Plus system equipped with a Waters 410 Differential Refractometer detector using an Aminex HPX87H Column (BioRad), operated in isocratic mode (5 mM H_2_SO_4_, 0.6 ml min^−1^).

### Quantification of intracellular hexose-phosphate

For intracellular metabolite quantification, 1–3 OD_750_ nm pellets culture samples were quickly collected by centrifugation (15 000 *g* for 30 s at 4 °C) and immediately frozen in liquid N_2_ for storage at –80 °C until metabolite extraction. Frozen pellets were carefully resuspended in 500 µl of ice-cold 70:30 methanol–chloroform mixture supplemented with 10 µM acetaminophen as internal standard. The samples were processed by five cycles of a 30 s vortex and cooling on liquid nitrogen. The vortexing treatment was repeated three times with 15 resting intervals at –20 °C between them. Next, 500 μl of ultrapure ice-cold water was added to the tubes and thoroughly mixed by vortexing. Samples were centrifuged (15 000 *g* for 5 min at 4 °C) and the upper polar phase was transferred to a new tube. To ensure maximum extraction, the apolar phase was subsequently mixed with another 250 μl of ultrapure ice-cold water and the polar phase was again collected and combined with the previous extraction. Samples were evaporated using a vacuum concentrator in a 4 °C room. Dried pellets were resuspended in 100 μl of ultrapure water and analyzed by LC-MS. Chromatographic separation was performed with an XSELECT HSS XP 150 mm×2.1 mm×2.5 μm clumn (Waters) in an Exion HPLC (Sciex) connected to a QTrap 6500+ (Sciex) operating in negative mode. Sample data were acquired and processed with Analyst and SciexOS software. For quantification of the total amount of the metabolites, different known concentrations of each standard were used.

### Cell lysates

Cell lysates were obtained after mechanical disruption with glass beads in 50 mM Tris–HCl (pH 8), 5 mM NaCl, and 1 mM PMSF by 10 cycles of 1 min vortexing/resting on ice. After centrifugation (15 000 *g* 20 min at 4 °C), the soluble fraction was recovered, and the protein was quantified with Bradford reagent (Biorad) using BSA (Sigma) as standard.

### Western blot

Equal amounts of protein from cell lysates were resolved by SDS–PAGE (10% acrylamide/bis-acrylamide) and transferred to nitrocellulose membranes (GE Healthcare) during 1 h at 0.8 mA cm^–2^. Membranes were blocked during 1 h in blocking solution [PBS-T solution (phosphate buffered saline with 1% Tween-20) and 5% non-fat milk powder (Applichem)] and immunoblotted overnight at 4 °C with antibodies against AGP (1:20 000), GlgA1 (1:5000), GlgA2 (1:5000) (Díaz-Troya *et al.*, 2014), PGM (1:50 000), PMM/PGM (1:20 000), or GroEL (1:75 000, Sigma-Aldrich) diluted in blocking solution. After incubation with primary antibody, membranes were rinsed four times for 10 min in PBS-T and then incubated for 1 h with horseradish peroxidase-conjugated secondary antibody (1:25 000 in blocking solution, Sigma). Membranes were then washed four times with PBS-T prior to chemiluminescent signal detection using Clarity™ western ECL Substrate (Biorad) and ImageQuant 800 imaging systems (Amersham). ImageQuant TL8.1 software was used for analysis.

### PGM and PMM/PGM quantification in cell extracts

Quantification of the absolute amount of PGM and PMM/PGM in WT soluble cell extracts was carried out by comparison with purified recombinant PGM and PMM/PGM. Serial known amounts of purified recombinant PGM or PMM/PGM and serial amounts of soluble cell extracts from the WT strain were resolved by SDS–PAGE, probed with antibodies against PGM or PMM/PGM, and analyzed with ImageQuant TL8.1 software as described above under ‘Western blot’.

For relative quantification of PMM/PGM levels in PMM/PGM-overexpressing strains compared with WT strains, equal amounts of soluble extracts from the different strains were resolved by SDS–PAGE, probed with antibodies against PMM/PGM, and analyzed as described above.

### Phosphoglucomutase activity in cell lysates

For phosphoglucomutase activity, quantification-coupled enzymatic assay following the reduction of NAD^+^ due to G6P oxidation was performed as described in Lindahl and Florencio (2003) with minor modifications. A total amount of 50–200 µg of extract proteins of the soluble fraction of *Synechocystis* strains was mixed in buffer with a final concentration of 100 mM Tris–HCl pH 8, 4 mM MgCl_2_, 2.5 mM NAD^+^, and 0.2 U of glucose-6-phosphate dehydrogenase from *Leuconostoc mesenteroides* (Sigma) in 200 µl. Absorbance at 340 nm was measured and the reaction was triggered by adding 4 mM G1P and 40 µM G16BP, and followed for 30 min at 30 °C. In parallel, as background control for every sample, the same reaction without G1P addition was performed and the changes in absorbance were subtracted from the reaction with G1P.

## Results

### PGM is 10 times more abundant than PMM/PGM in *Synechocystis*

As previously indicated, *Synechocystis* has two enzymes with phosphoglucomutase activity, PGM and PMM/PGM. Using specific antibodies raised against these two proteins, we could detect both by western blot under our standard growth conditions (cultures under constant 50 µE m^−2^ s^−1^ illumination and bubbled with 1% CO_2_-supplemented air). Quantification of these proteins in cell extracts (as described in the Materials and methods) indicated that PGM was ~10-fold more abundant than PMM/PGM (0.75 ± 0.03 ng of PGM and 0.07 ± 0.02 ng of PMM/PGM per µg of soluble cell extract).

**Fig. 2.**
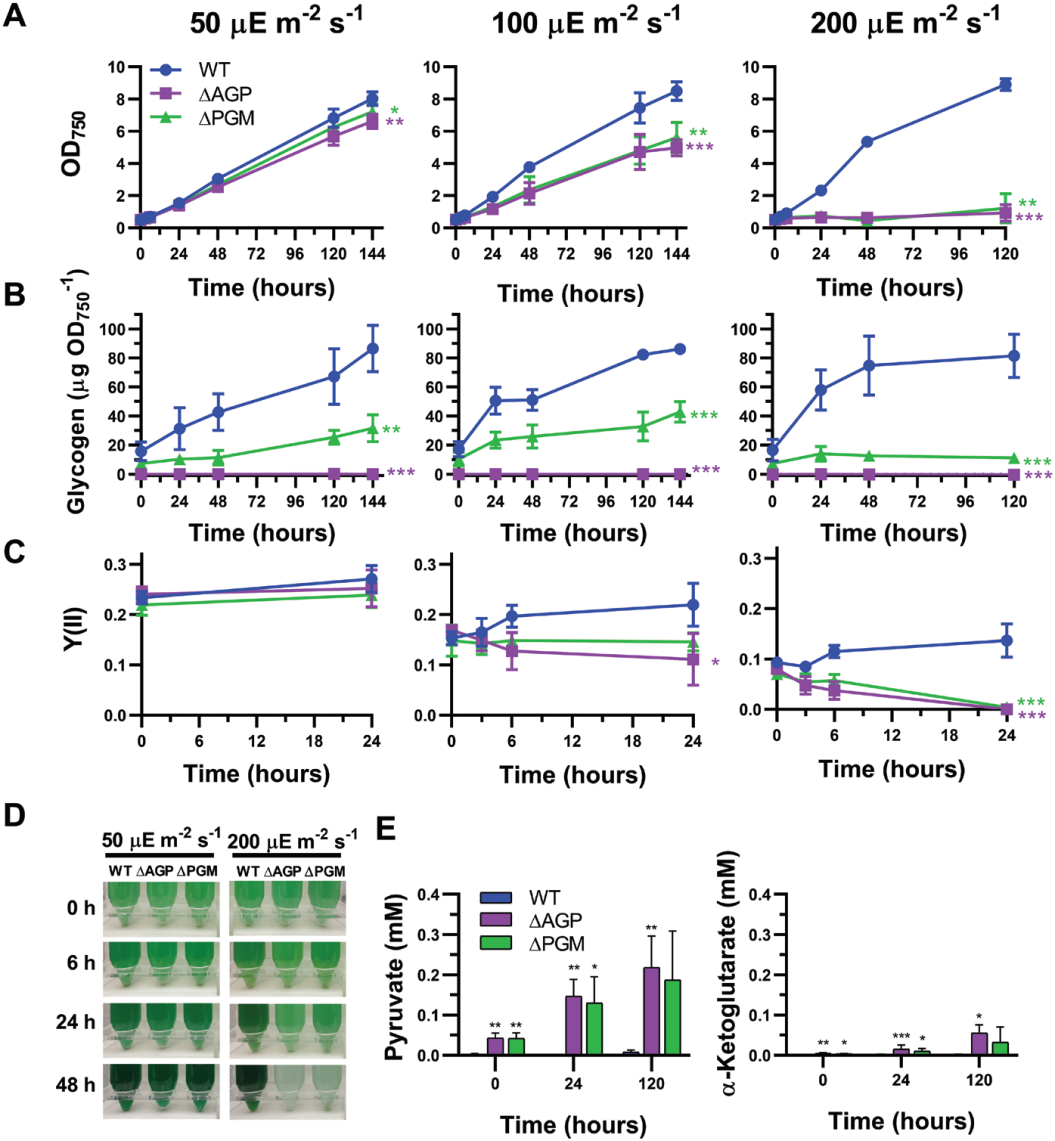
Characterization of the phenotype of the ∆PGM strain in response to high light exposure. WT, ∆AGP, and ∆PGM cultures bubbled with 1% CO_2_-supplemented air at 50 µE m^−2^ s^−1^ light intensity were used to inoculate cultures at an initial OD_750 nm_ of 0.5. These cultures were grown at different light intensities (50, 100, and 200 µE m^−2^ s^−1^) for 6 d. (A) Growth curves of the WT, ∆AGP, and ∆PGM strains at 50, 100, and 200 µE m^−2^ s^−1^ measured as optical density at 750 nm. (B) Glycogen content of the WT, ∆AGP, and ∆PGM strains at 50, 100, and 200 µE m^−2^ s^−1^. Experiments at 200 µE m^−2^ s^−1^ were terminated after 5 d because ∆AGP and ∆PGM had whitened. (C) Effective quantum yields of energy conversion in PSII [Y(II)] of the WT, ∆AGP, and ∆PGM strains at 50, 100, and 200 µE m^−2^ s^−1^ measured by PAM fluorometry with an actinic light of 50, 100, and 200 µE m^−2^ s^−1^, respectively, during the first 48 h of the experiment. (D) Photographs of WT, ∆AGP, and ∆PGM cultures after 0, 6, 24, and 48 h growing at 50 µE m^−2^ s^−1^ or 200 µE m^−2^ s^−1^. (E) Concentration of pyruvate and α-ketoglutarate released to the culture media by the WT, ∆AGP, and ∆PGM cells after 0, 24, and 120 h growing at 100 µE m^−2^ s^−1^. Data are means ±SD from four (A–C) or three (E) biological replicates. In (A–C), significant differences in the last time point compared with the value of the WT strain were determined using paired two-tailed Student’s *t*-test. The color of the asterisks matches the color of the corresponding strain. In (E), significant differences compared with the value of the WT strain were determined using unpaired two-tailed Student’s *t*-test: *P<0.05, **P<0.01, ***P<0.001.

To better understand the role of these two proteins in glycogen metabolism, we tried to generate mutants lacking PGM and PMM/PGM. Consistent with previously described results (Liu *et al.*, 2013), a mutant lacking the bifunctional enzyme PMM/PGM could not be segregated (∆PMM* strain), suggesting that PMM/PGM has an essential role in *Synechocystis*. In contrast, the deletion of *sll0726* (coding for PGM) was easily segregated, generating the ∆PGM strain (Supplementary Fig. S1). The ∆PGM mutant accumulated moderate amounts of glycogen in all growth conditions tested, including cultures bubbled with CO_2_-supplemented air or non-bubbled flask cultures in media with different concentrations of NaHCO_3_ (Fig. 2B; Supplementary Fig. S2). After 6 d, the glycogen accumulated in the ∆PGM strain was 37% of that in the WT under our standard growth conditions (constant 50 µE m^−2^ s^−1^ illumination and 1% CO_2_-supplemented air) (Fig. 2B). This indicates that, in addition to PGM, there is another protein in *Synechocystis* with phosphoglucomutase activity, probably PMM/PGM, capable of providing G1P for glycogen synthesis, although less efficiently than PGM.

Glycogen has been proposed to act not only as carbon storage but also as an energy buffer to maintain energy homeostasis in the cell (Cano *et al.*, 2018). For this reason, we characterized the phenotype of the ∆PGM strain to analyze the effect of a limited carbon flux to and from glycogen under different growth conditions. We have chosen contexts in which the buffer role of glycogen could be more relevant, which requires increased accumulation or mobilization of carbon storage, or increased availability of ADP-glucose, such as high light, diel cycles, nitrogen deprivation, or salt stress.

### PGM is required to survive under high light exposure

Under high light growth conditions, lack of glycogen synthesis causes overflow of organic acids to dissipate excess energy and negatively affects cell performance (Carrieri *et al.*, 2012; Gründel *et al.*, 2012; Cano *et al.*, 2018). This suggests that limitations in carbon flux to glycogen in the ∆PGM strain could also negatively affect cell performance at higher light intensities. To test this, we analyzed the phenotype of the ∆PGM strain grown under different light intensities and compared it with the WT and the glycogen-less ∆AGP strain, which lacks the enzyme ADP-glucose pyrophosphorylase (Fig. 2). Cells grown under constant 50 µE m^−2^ s^−1^ illumination were maintained in this situation or transferred to 100 µE m^−2^ s^−1^ or 200 µE m^−2^ s^−1^. The WT and mutant strains showed similar growth rates at 50 µE m^−2^ s^−1^. However, the growth of the ∆AGP and ∆PGM strains was negatively affected at 100 µE m^−2^ s^−1^. When transferred to 200 µE m^−2^ s^−1^, the growth of the mutant strains was completely suppressed and the cultures turned white and died after 48–72 h, while the growth of the WT strain was not affected (Fig. 2A, D).

Glycogen content was also affected by light intensity (Fig. 2B). The WT strain accumulated up to 90 µg/OD_750 nm_ independently of the light conditions, but this accumulation was faster at 100 µE m^−2^ s^−1^ and 200 µE m^−2^ s^−1^ than at 50 µE m^−2^ s^−1^ (Fig. 2B). After 6 d, the accumulation of glycogen in the ∆PGM strain at 100 µE m^−2^ s^−1^ was 36% higher than at 50 µE m^−2^ s^−1^, representing 49% of the glycogen content in the WT under the same conditions. However, the loss of viability in the ∆PGM strain after 5 d at 200 µE m^−2^ s^−1^ correlated with a decrease in glycogen content compared with its content at 50 µE m^−2^ s^−1^ (12.7 µg/OD_750 nm_ versus 31.6 µg/OD_750 nm_, *P*<0.05) or 100 µE m^−2^ s^−1^ (43.0 µg/OD_750 nm_, *P*<0.01; unpaired two-tailed Student’s *t*-test) (Fig. 2B). As expected, no glycogen was detected in the ∆AGP strain in any condition (Fig. 2B).

The shift to higher light intensity affected the photosynthetic performance of the WT and mutant strains. We used PAM to measure effective quantum yields of energy conversion in PSII [Y(II)] of the cultures during the first 48 h after being transferred to the different growth light intensities (Fig. 2C). Measurements were carried out using the same light intensity used to culture the strains (50, 100, or 200 µE m^−2^ s^−1^). Y(II) at 50 µE m^−2^ s^−1^ was similar for the three strains. An increase in the light intensity immediately caused a similar decrease in the Y(II) in the WT and mutant strains. However, only the WT strain was able to adapt to the higher light intensities. After 24 h at 100 µE m^−2^ s^−1^ and 200 µE m^−2^ s^−1^, the WT strain recovered Y(II) values that were 80% and 51%, respectively, of the value at 50 µE m^−2^ s^−1^ (Fig. 2C). However, after 24 h at 100 µE m^−2^ s^−1^, Y(II) of the ∆AGP and ∆PGM strains was still 42% and 65%, respectively, of Y(II) at 50 µE m^−2^ s^−1^. The effect of high light on photosynthetic performance was more dramatic at 200 µE m^−2^ s^−1^, with a Y(II) value of zero for both mutant strains after 24 h (Fig. 2C). Furthermore, unlike the WT, the ∆AGP and ∆PGM strains secreted similar levels of the organic acids pyruvate and α-ketoglutarate at 100 µE m^−2^ s^−1^ (Fig. 2E). All this indicates that the reduced carbon flux between G1P and G6P present in the ∆PGM strain, which only allows the accumulation of limited levels of glycogen, was not sufficient to buffer the excess energy at higher light intensities.

### Lack of PGM causes growth retardation under short-day conditions

**Fig. 3.**
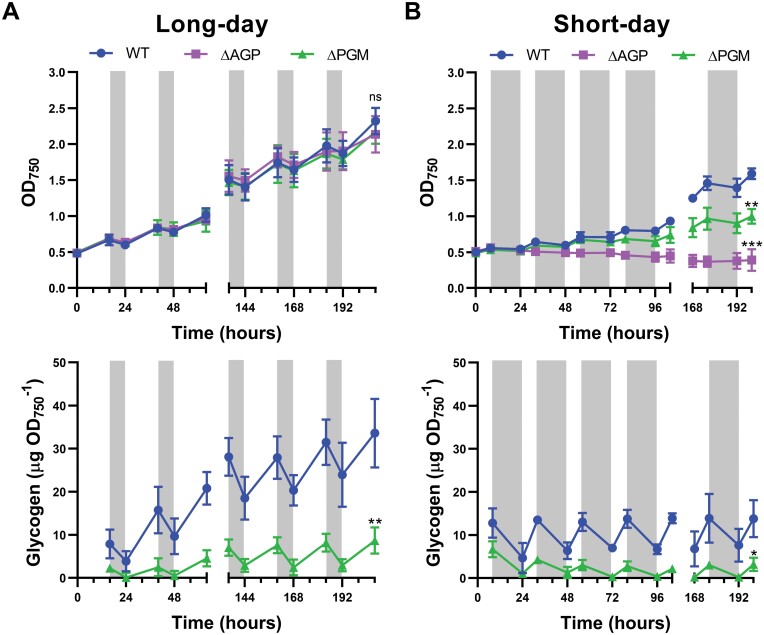
Growth curves and glycogen content of the ∆PGM strain cultivated under long-day and short-day diel regimes. WT, ∆AGP, and ∆PGM cultures inoculated at an OD_750 nm_ of 0.5 in Erlenmeyer flasks were cultivated under (A) long-day (16 h light/8 h dark) or (B) short-day (8 h light/16 h dark) regimes at 40 µE m^−2^ s^−1^ for 9 d. White and gray areas represent light and dark periods, respectively. Growth (measured as OD_750_) and glycogen content were determined at the indicated time points. Data are means ±SD from three biological replicates. Significant differences in OD_750 nm_ or glycogen content at the end of the experiment compared with the WT strain were determined using paired two-tailed Student’s *t*-test: *P<0.05, **P<0.01, ***P<0.001. The color of the asterisks matches the color of the corresponding strain.

Glycogen also plays a significant role during light–dark periods, and the growth and viability of glycogen-less mutants are affected under these conditions (Gründel *et al.*, 2012; Welkie *et al.*, 2019). We analyzed the effect of limited synthesis of glycogen in the ∆PGM strain under long-day (16 h light/8 h dark) and short-day (8 h light/16 h dark) regimes (Fig. 3). Similarly to the WT, glycogen content in the ∆PGM strain increased during light and decreased in the dark in both regimes, although to a lesser extent than in the WT. Despite reduced amounts of glycogen in the ∆PGM strain compared with the WT, both grew quite similarly under a long-day regime (Fig. 3A). Surprisingly, under these conditions, the ∆AGP strain also showed a growth rate close to that of the WT and ∆PGM strains in the absence of detectable glycogen (Fig. 3A). Thus, after 200 h under a long-day regime, the three strains reached a similar final OD_750_ (OD _750 nm_=2.2 ± 0.1) (Fig. 3A). In contrast, after 200 h growth in a short-day regime, the ∆PGM culture reached a lower final OD_750 nm_ than the WT, while the growth of the ∆AGP strain was completely abolished (Fig. 3B), indicating the importance of glycogen storage during longer dark periods.

### PGM is required for an adequate response to nitrogen deprivation

**Fig. 4.**
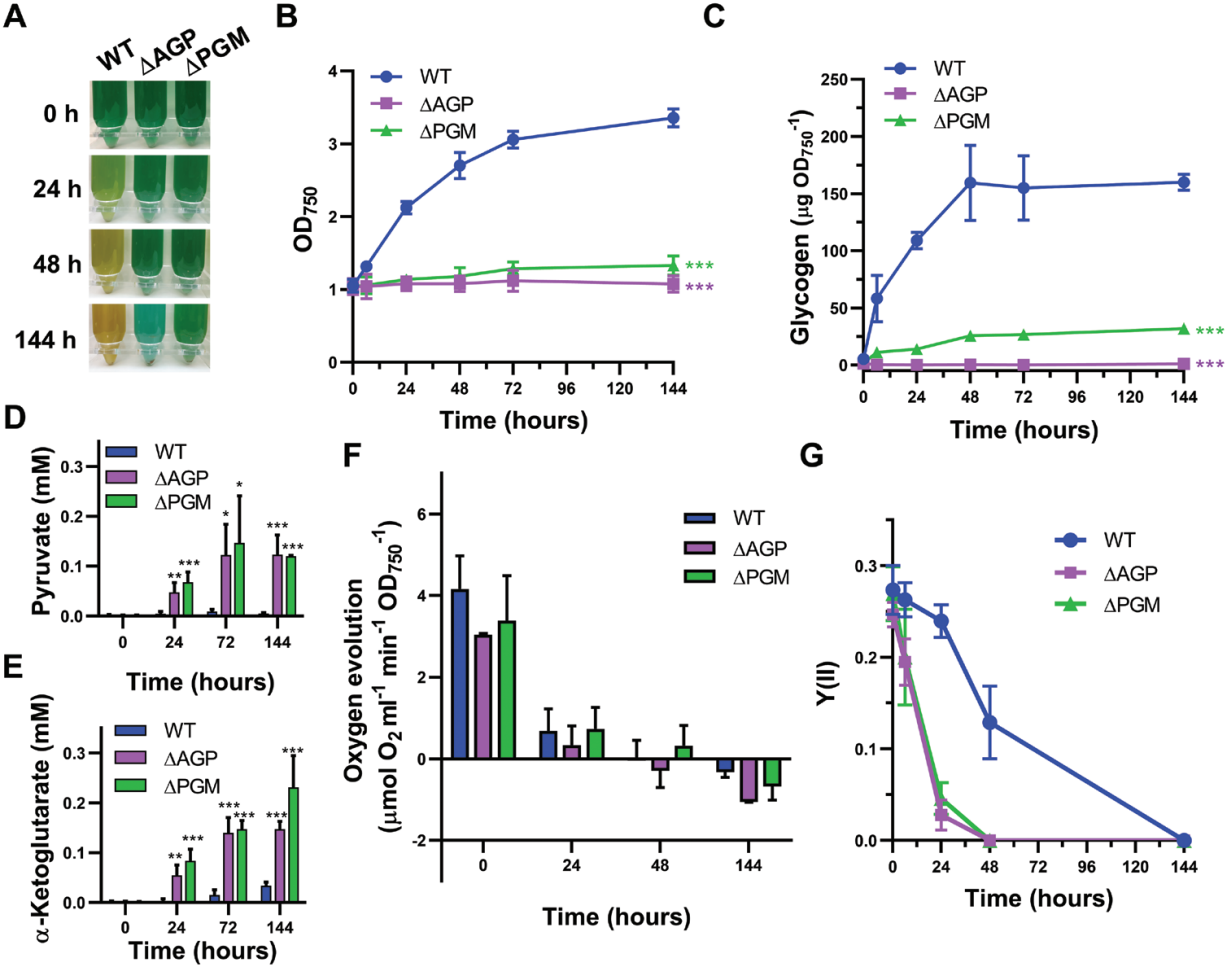
Characterization of the phenotype of the ∆PGM strain in response to nitrogen deprivation. WT, ∆AGP, and ∆PGM cells were grown in BG11C to mid-exponential growth phase, washed twice in nitrogen-free medium, and transferred to nitrogen-free medium at an OD_750 nm_ of 1. (A) Photographs of the WT, ∆AGP, and ∆PGM cultures 0, 24, 48, and 144 h after nitrogen removal. (B) Growth curves of the WT, ∆AGP, and ∆PGM strains cultivated in nitrogen-free medium measured as optical density at 750 nm. (C) Glycogen content of the WT, ∆AGP, and ∆PGM strains at different time points after nitrogen removal. Concentration of extracellular (D) pyruvate and (E) α-ketoglutarate in WT, ∆AGP, and ∆PGM cultures 0, 24, 48, and 144 h after nitrogen removal. (F) Oxygen evolution of the WT, ∆AGP, and ∆PGM cultures at different times after nitrogen removal measured by a Clark-type electrode at the growth light intensity (50 µE m^−2^ s^−1^). (G) PSII quantum yields [Y(II)] in WT, ∆AGP, and ∆PGM cultures measured by PAM fluorometry at the growth light intensity (50 µE m^−2^ s^−1^) 0, 24, 48, and 144 h after nitrogen removal. Data are means ±SD from four (B, D–G) or three (C) biological replicates. In (B and C), significant differences in OD_750 nm_ or glycogen content at 144 h after nitrogen removal compared with the WT strain were determined using paired two-tailed Student’s *t*-test. The color of the asterisks matches the color of the corresponding strain. In (D and E), significant differences compared with the value of the WT strain at the same temporal point were determined using unpaired two-tailed Student’s *t*-test: *P<0.05, **P<0.01, ***P<0.001.

In addition to light-dependent phenotypes, the response to nitrogen deficiency is also altered in glycogen-less mutants (Gründel *et al.*, 2012; Jackson *et al.*, 2015; Díaz-Troya *et al.*, 2020). Thus, WT cells during nitrogen deficiency conditions accumulate large amounts of glycogen and degrade their phycobiliproteins to recycle the nitrogen present in them and adjust to the new nutritional situation. This causes the loss of their characteristic blue-green color, that turns into a yellow color, a process known as bleaching or chlorosis (Forchhammer and Schwarz, 2019). Mutants unable to accumulate glycogen do not degrade their phycobiliproteins and compromise their viability in nitrogen deprivation conditions (Gründel *et al.*, 2012). As shown in Fig. 4, the lack of PGM clearly impaired the adequate adaptation to nitrogen deficiency. Thus, similarly to the ∆AGP strain, when the ∆PGM strain was transferred to a medium without nitrogen the growth of the culture ceased immediately and degradation of phycobiliproteins did not occur (Fig. 4A, B; Supplementary Fig. S3A). This happened even though the ∆PGM strain accumulated glycogen, although in much lower amounts than the WT (32 µg/OD_750_ versus 160 µg/OD_750_) (Fig. 4C). Under these conditions, carbon flux was clearly limited in the ∆PGM strain, causing excretion of organic acids at levels similar to those of the ∆AGP strain (Fig. 4D, E).

One of the effects of nitrogen deficiency is the decrease in photosynthetic activity (Krasikov *et al.*, 2012; Ogawa and Sonoike, 2016). We observed this decrease in both the WT and mutant strains, despite the presence of phycobiliproteins in the latter (Fig. 4F). Therefore, net oxygen evolution was residual in the WT, ∆AGP, or ∆PGM strains 48 h after nitrogen removal (Fig. 4F). To investigate how photosynthesis is affected in the ∆PGM strain in this situation, we tested its photosynthetic performance by using PAM analysis (Fig. 4G; Supplementary Fig. S3C). In the WT, nitrogen deprivation produced a 45% decrease in Y(II) after 48 h compared with nitrogen-replete conditions, and after 6 d this value was zero (Fig. 4G). The WT showed a decrease in both basal fluorescence (*F*_o_) and maximum fluorescence (*F*_m_) due to degradation of the phycobilisomes (Supplementary Fig. S3C). Y(II) values of the ∆AGP and ∆PGM strains in nitrogen-repleted medium were equivalent to that of the WT (Fig. 4G). However, nitrogen deprivation induced a faster Y(II) decrease in the ∆AGP and ∆PGM strains than in the WT, with Y(II) values close to zero at 48 h (Fig. 4G). Furthermore, in contrast to the WT, *F*_o_ increased in the ∆PGM strain (Supplementary Fig. S3C), suggesting an increase in free phycobilisomes (Acuña *et al.*, 2016; Stirbet *et al.*, 2019).

An adequate response to nitrogen deprivation is essential for survival. Under these conditions, *Synechocystis* cells enter a dormant-like state in which they can survive for at least 1 month. They are able to recover vegetative growth once nitrogen is available again (Neumann *et al.*, 2021). However, mutants unable to accumulate glycogen not only have a non-bleaching phenotype, but also exhibit decreased viability when faced with nitrogen deprivation (Gründel *et al.*, 2012; Doello *et al.*, 2022). Under our growth conditions, after 15 d of nitrogen starvation, ∆AGP and ∆PGM cultures had turned white, lost photosynthetic pigments, and the viability of the cultures was severely compromised (Supplementary Fig. S4).

**Fig. 5.**
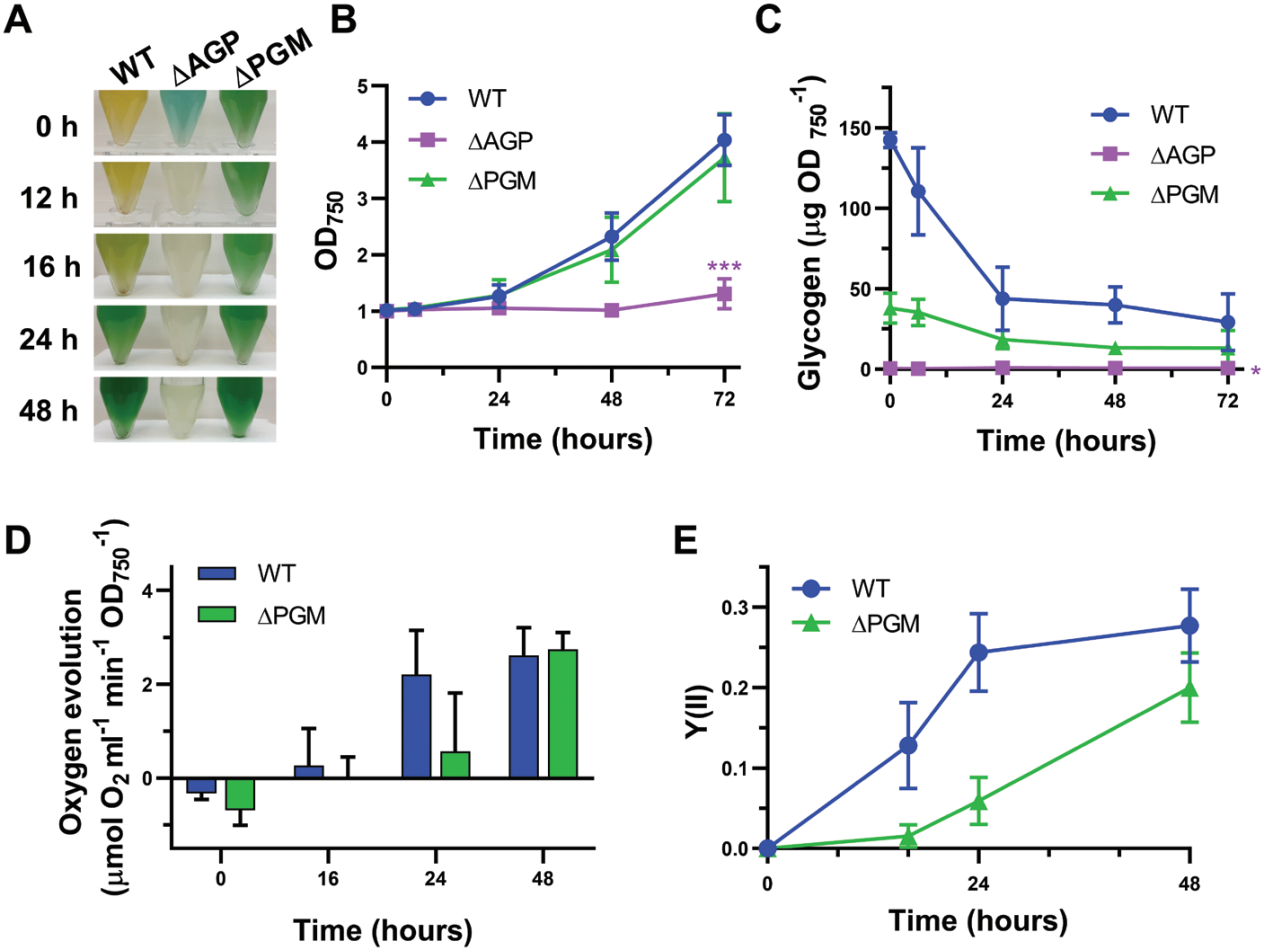
Growth, glycogen content, and photosynthetic characterization of the strain ∆PGM after nitrogen replenishment. Cells of the WT, ∆AGP, and ∆PGM strains cultured in nitrogen-free medium for 6 d were collected and transferred to nitrogen-replete medium at an optical density at 750 nm of 1. (A) Photographs of the WT, ∆AGP, and ∆PGM cultures at different time points after nitrogen replenishment. (B) Growth curves of the WT, ∆AGP, and ∆PGM cultures after nitrogen replenishment. (C) Evolution of the glycogen content of the WT, ∆AGP, and ∆PGM cultures over the course of 3 d after nitrogen replenishment. (D) Oxygen evolution of the WT and ∆PGM cultures at different times after nitrogen replenishment measured by a Clark-type electrode at the growth light intensity (50 µE m^−2^ s^−1^). (E) PSII quantum yields [Y(II)] in WT and ∆PGM cultures measured by PAM fluorometry at the growth light intensity (50 µE m^−2^ s^−1^) at different times after nitrogen replenishment. Data are means ±SD from four biological replicates. In (B and C), significant differences in OD_750 nm_ or glycogen content at the end of the experiment compared with the WT strain were determined using paired two-tailed Student’s *t*-test. The color of the asterisks matches the color of the corresponding strain. *P<0.05, **P<0.01, ***P<0.001.

Recovery from this dormancy state induced by nitrogen deprivation requires mobilization of glycogen to provide energy to restore the translational machinery, ATP synthesis, and nitrate assimilation, and finally for synthesis of the photosynthetic apparatus (Neumann *et al.*, 2021). To investigate whether mobilization of the limited glycogen accumulated in the ∆PGM strain confers an advantage over the glycogen-less ∆AGP strain, we analyzed their recovery after the addition of nitrate as a nitrogen source (Fig. 5). As mentioned above, both strains showed reduced viability after long periods of nitrogen starvation (Supplementary Fig. S4). Therefore, we limited the length of the nitrogen starvation period to 6 d. After this time, the WT had fully degraded their phycobiliproteins and no net oxygen evolution was detected in the WT or the mutant strains (Supplementary Fig. S3A; Fig. 4F). Under these conditions, transfer of the cells to nitrogen-replete medium induced a fast mobilization of the glycogen in the WT cells, which recovered their blue-green pigmentation and resumed growth (Fig. 5A–C; Supplementary Fig. S3B). In contrast, nitrogen addition did not prevent whitening of the ∆AGP cells, which were unable to recover after 6 d of nitrogen deprivation. Before nitrogen replenishment, both ∆PGM and ∆AGP maintained their blue-green color. After addition of nitrate, the ∆PGM strain mobilized its glycogen reserves, although to a lesser extent than the WT, both in total amount per unit of optical density at 750 nm (98 µg/OD_750_ and 19 µg/OD_750_ in 24 h for the WT and ∆PGM strains, respectively) and as a percentage of the initial glycogen amount (70% and 51% for the WT and ∆PGM strains, respectively). However, this was enough to resume its growth at a similar rate to that of the WT (Fig. 5A–C). Differences were also found in the photosynthetic performance of the WT and ∆PGM strains after nitrate addition (Fig. 5D, E), with a faster recovery of Y(II) in the WT than in the ∆PGM strain, although the ∆PGM cells had not degraded their photosynthetic pigments. However, the slower recovery of O_2_ evolution of the ∆PGM strain was not statistically significant (Fig. 5D, E).

### Lack of PGM does not impair resistance to salt stress

**Fig. 6.**
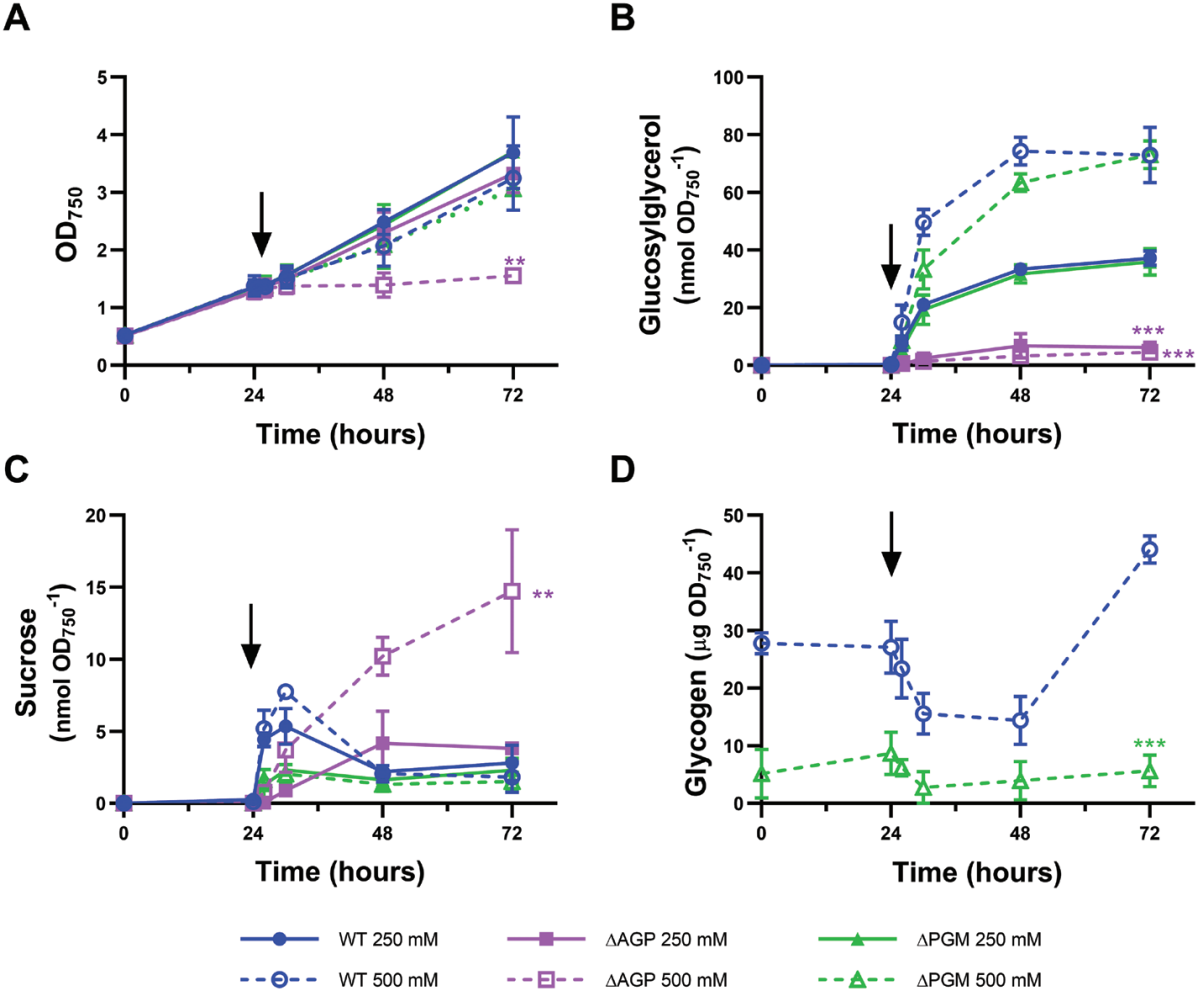
Effect of NaCl addition on growth and osmolyte and glycogen content of the WT, ∆AGP, and ∆PGM strains. WT, ∆AGP, and ∆PGM cells were cultured in standard medium without additional supplementation with NaCl for 24 h and then 250 mM or 500 mM NaCl was added. (A) Growth curves of the WT, ∆AGP, and ∆PGM cultures. Intracellular content of (B) glucosylglycerol and (C) sucrose of the WT, ∆AGP, and ∆PGM cultures during the experiment. (D) Glycogen content of the WT and ∆PGM cultures before and after the addition of 500 mM NaCl. Vertical arrows indicate NaCl addition. Data are means ±SD from three biological replicates. Significant differences at the end of the experiment compared with the WT strain at the same NaCl concentration were determined using unpaired two-tailed Student’s *t*-test. The color of the asterisks matches the color of the corresponding strain. *P<0.05, **P<0.01, ***P<0.001.

Salt tolerance is one of the characteristics that could be affected by a limitation in the G6P–G1P interconversion, as G1P is required for the synthesis of osmolytes in *Synechocistis* (Kirsch *et al.*, 2019). The synthesis and accumulation of the osmolytes sucrose and, in particular, glucosylglycerol (GG) allows *Synechocystis* cultures to tolerate moderate salinities (up to 1 M NaCl) (Mackay *et al.*, 1984; Kirsch *et al.*, 2019). Sucrose is synthesized from fructose-6-phosphate and UDP-glucose, and GG from glycerol-3-phosphate and ADP-Glc (Kirsch *et al.*, 2019) (Fig. 1). The supply of both UDP-glucose and ADP-Glc is based on G1P and, thus, on phosphoglucomutase activity. In this regard, mutants lacking AGP present a salt-sensitive phenotype due to their inability to synthesize ADP-Glc and therefore GG (Miao *et al.*, 2003). To analyze the influence of limitation on G6P–G1P interconversion on salt tolerance, the WT, ∆AGP, and ∆PGM strains were cultured in medium without additional supplementation of NaCl for 24 h and then NaCl was added to reach a final concentration of 250 mM or 500 mM (Fig. 6). In the WT, this induced the accumulation of GG up to 37 nmol/OD_750_ and 73 nmol/OD_750_ for 250 mM and 500 mM NaCl, respectively, and a fast and transient accumulation of sucrose (Fig. 6A–C). As expected, the ∆AGP strain did not accumulate GG. However, the accumulation of sucrose allowed the culture to grow similarly to the WT at 250 mM NaCl but not at 500 mM NaCl (Fig. 6A–C). In the case of the ∆PGM strain, although the maximum accumulation of sucrose was lower than in the WT, GG reached amounts similar to those of the WT, but with slightly delayed kinetics at 500 mM NaCl. However, these alterations in the accumulation of osmolytes did not affect the growth of the ∆PGM strain at 250 mM or 500 mM NaCl (Fig. 6A). Interestingly, the addition of NaCl induced the transient mobilization of glycogen in both the WT and ∆PGM strains (Fig. 6D). Glycogen accumulation resumed after 48–72 h, when the amount of GG stabilized at its maximum level in both strains (Fig. 6B).

### The overexpression of PMM/PGM compensates for the lack of PGM

**Fig. 7.**
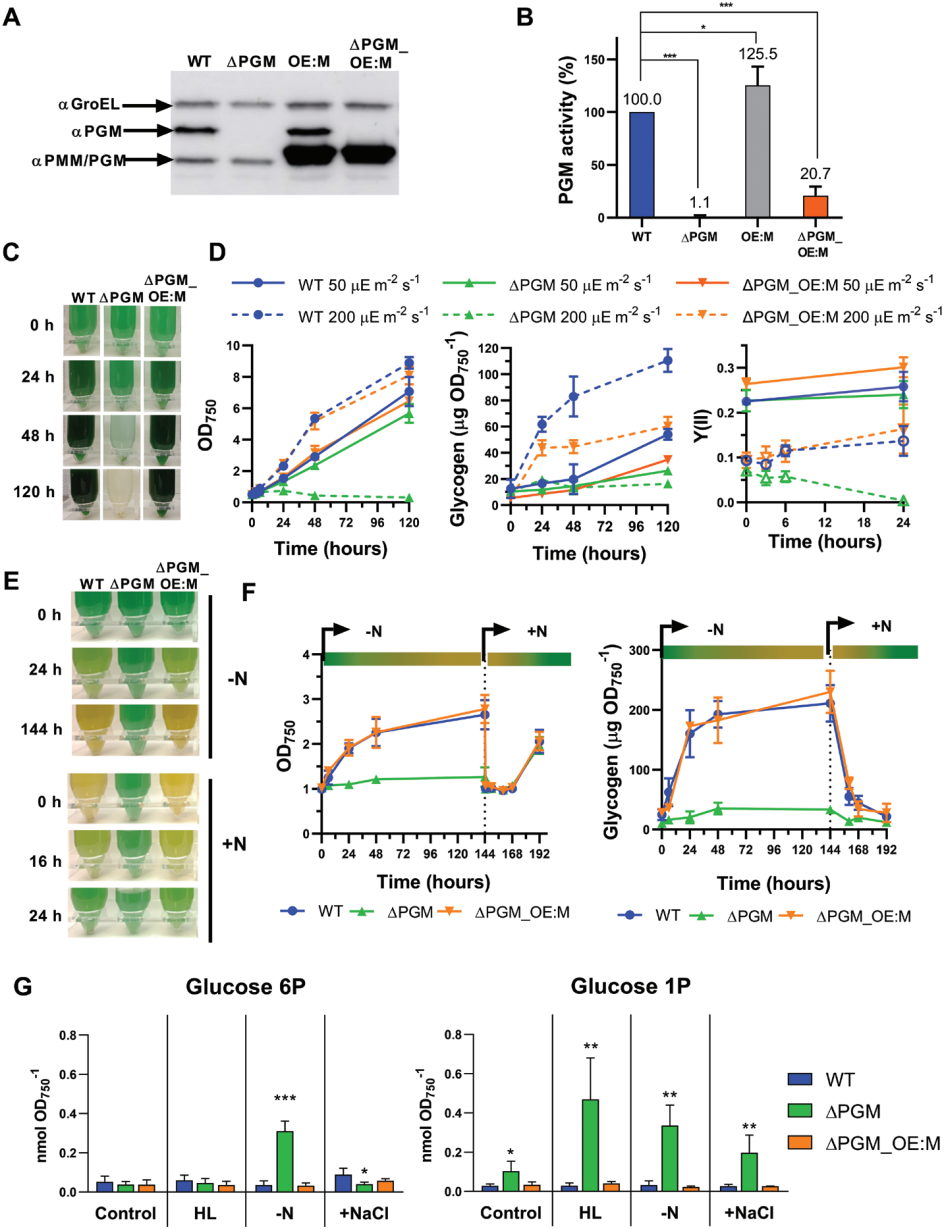
PMM/PGM overexpression and characterization of the ∆PGM_OE:M strain in response to high light and nitrogen deprivation. (A and B) Analysis of PMM/PGM overexpression. (A) Soluble extracts from samples of the WT, ∆PGM, OE:M, and ∆PGM_OE:M cultures grown to mid-exponential phase under standard growth conditions were analyzed by western blot with antibodies against PGM and PMM/PGM. GroEL was used as loading control. A 10 µg aliquot of protein was loaded in each lane. (B) Phosphoglucomutase activity was assayed in soluble extracts from WT, ∆PGM, OE:M, and ∆PGM_OE:M cells at mid-exponential phase under standard growth conditions. Values were referenced to WT phosphoglucomutase activity. (C and D) Effect of high light. WT, ∆PGM, and ∆PGM_OE:M cells grown under 50 µE m^−2^ s^−1^ were used to inoculate cultures at 50 µE m^−2^ s^−1^ or 200 µE m^−2^ s^−1^. (C) Photographs of the WT, ∆PGM, and ∆PGM_OE:M cultures grown at 200 µE m^−2^ s^−1^. (D) Growth curves, glycogen content, and Y(II) of the WT, ∆PGM, and ∆PGM_OE:M cultures grown at 50 µE m^−2^ s^−1^ or 200 µE m^−2^ s^−1^. (E and F) Effect of nitrogen deprivation. WT, ∆PGM, and ∆PGM_OE:M cultures grown in BG11C to mid-exponential phase were washed to remove nitrogen and transferred to nitrogen-free medium (BG11_0_C) at an optical density at 750 nm of 1. After 6 d, cells were again transferred to standard BG11C media at 1 OD_750_. (E) Representative photographs of the WT, ∆PGM, and ∆PGM_OE:M cultures during nitrogen deprivation (marked as –N on the right of the panels) and after nitrogen replenishment (marked as +N). (F) Growth curves and glycogen content of the WT, ∆PGM, and ∆PGM_OE:M cultures during the nitrogen deprivation and nitrogen replenishment periods (represented by a green to yellow bar and a yellow to green bar, respectively). Vertical dotted lines indicate the transfer of the cells to nitrogen-replete media at 1 OD_750_. (G) Content of glucose-1-phosphate and glucose-6-phosphate. WT, ∆PGM, OE:PMM, and ∆PGM_OE:PMM strains were maintained under standard growth conditions, nitrogen starved, exposed to high light (200 µE), or supplemented with 500 mM NaCl for 24 h. Cells were collected and G1P and G6P contents were measured. Data are means ±SD from four (B, F, G) or three (D) biological replicates. In (B and G), significant differences compared with the WT under the same growth conditions were determined using unpaired two-tailed Student’s *t*-test. *P<0.05, **P<0.01, ***P<0.001.

As indicated above, PMM/PGM is 10 times less abundant in the cell than PGM. Furthermore, *in vitro* characterization of recombinant enzymes indicates that the phosphoglucomutase activity of PMM/PGM is also 10 times lower than that of PGM (Liu *et al.*, 2013). The above data also suggest that the low phosphoglucomutase activity of PMM/PGM is sufficient to support reduced glycogen synthesis in the ∆PGM strain, so we hypothesized that PMM/PGM could functionally compensate for the lack of PGM if it is expressed at higher levels. To test this, we constructed mutant strains overexpressing the *slr1334* gene (coding for PMM/PGM) under the control of the strong constitutive promoter P_cpcB_ (Zhou *et al.*, 2014; García-Cañas *et al.*, 2021) in the WT and ∆PGM strains, resulting in the OE:M and ∆PGM*_*OE:M strains, respectively. These two strains overexpressed PMM/PGM (27.5 ± 5.0 times compared with the WT strain) (Fig. 7A). This resulted in a 25% increase in phosphoglucomutase activity in cell extracts from the OE:M strain compared with the WT and in a 20 times increase in the phosphoglucomutase activity in cell extracts of the ∆PGM*_*OE:M strain compared with its parental strain ∆PGM under standard growth conditions (Fig. 7B; Supplementary Fig. S5A). Similar phosphoglucomutase activity levels were obtained when these strains were exposed to high light (200 µE m^−2^ s^−1^), nitrogen deprived, or salt stressed (500 mM) for 24 h (Supplementary Fig. S5B). Protein levels of other enzymes involved in glycogen synthesis (AGP, GlgA1, and GlgA2) were not altered (Supplementary Fig. S5C).

Overexpression of PMM/PGM in the ∆PGM strain (∆PGM_OE:M strain) suppressed the phenotypes associated with the lack of PGM (Fig. 7C–F; Supplementary Fig. S6). In contrast to the ∆PGM strain, the ∆PGM_OE:M strain was not sensitive to high light (Fig. 7C, D). At 200 µE m^−2^ s^−1^, growth and photosynthetic performance of the ∆PGM_OE:M strain were similar to those of the WT and its glycogen content was higher than that of the ∆PGM strain (Fig. 7C, D). The phosphoglucomutase activity of PMM/PGM was also enough to sustain growth and glycogen accumulation during diel cycles (Supplementary Fig. S6). Glycogen levels of the ∆PGM_OE:M strain were similar to those of the WT in both long- and short-day conditions (Supplementary Fig. S6). The growth retardation shown by the ∆PGM strain under the short-day regime was also abolished in the ∆PGM_OE:M strain (Supplementary Fig. S6). The overexpression of PMM/PGM also recovered the response to nitrogen starvation (Fig. 7E, F). Therefore, when nitrogen was eliminated from the medium, the ∆PGM_OE:M cells degraded their phycobiliproteins and the culture increased its optical density 1.5 times and accumulated glycogen up to 205 µg/OD_750 nm_. (Fig. 7E, F). Furthermore, when a nitrogen source was provided to the chlorotic ∆PGM_OE:M culture, we observed a fast mobilization of glycogen, cells recovered their blue-green color, and the culture resumed growth (Fig. 7E, F). The overexpression of PMM/PGM in a WT background (OE:M strain) did not cause any detrimental effect under any of the assayed growth conditions (Supplementary Fig. S7A).

Finally, we measured G1P and G6P levels in the WT, ∆PGM, and ∆PGM*_*OE:M strains (Fig. 7G). Surprisingly, under control growth conditions, G1P levels, but not G6P levels, were higher (3.6 times) in the ∆PGM strain than in the WT. In contrast, in the ∆PGM*_*OE:M strain, both G1P and G6P levels were similar to those in the WT. This indicates that, although not phenotypically evident, lack of PGM is already causing metabolic disturbances under standard growth conditions and that overexpression of PMM/PGM compensates this imbalance. G1P and G6P were also measured after 24 h of exposure to high light, nitrogen deprivation, or addition of NaCl to the media (Fig. 7G). Under these conditions, G1P levels in the ∆PGM strain were 13, 10, and 7.5 times higher, respectively, than in the WT. In contrast, G6P levels were different in the ∆PGM strain depending on the growth conditions assayed. Thus, under high light conditions, G6P levels in the ∆PGM strain were similar to those in the WT. However, when the cultures were nitrogen deprived, G6P levels in the ∆PGM strain were nine times higher than in the WT. Salt stress resulted in a 50% decrease in G6P in the ∆PGM strain compared with the WT. In every case, overexpression of PMM/PGM restored G1P and G6P levels (Fig. 7G). This unexpected increase in G1P levels in the ∆PGM strain could suggest a lower efficiency of PMM/PGM in the synthesis of G6P from G1P, although other reasons cannot be discarded. In addition, the fact that the ∆PGM strain accumulates lower levels of glycogen, despite those higher G1P levels, suggests an effect on the regulation of AGP activity. In the case of G6P increase under nitrogen deprivation, it could reflect an inability of PMM/PGM to support the high carbon flux towards glycogen synthesis under this condition.

### PMM/PGM is an essential protein in *Synechocystis*

**Fig. 8.**
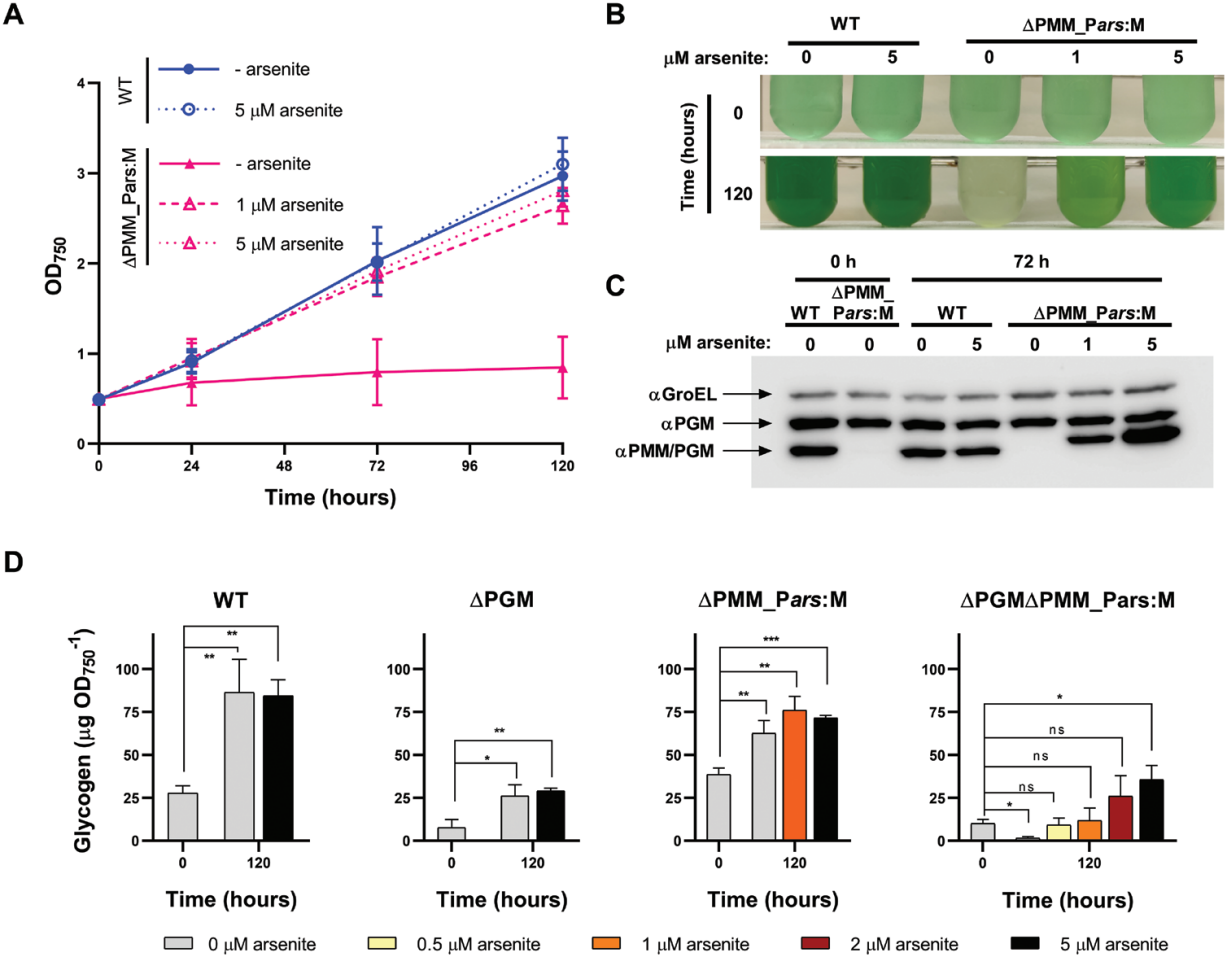
Characterization of the strains with regulated expression of PMM/PGM. WT and ∆PMM_P*ars*:M cells were cultured in the absence of arsenite for 6 d to ensure low levels of PMM/PGM in the ∆PMM_P*ars*:M and then transferred to medium without arsenite or with 1 µM or 5 µM arsenite at an OD_750_ of 1. Cultures were analyzed during the next 5 d of cultivation under standard growth conditions. (A) Growth curves of the cultures. (B) Photographs of the cultures at point 0 h and 120 h at the indicated arsenite concentrations. (C) Samples of the WT and ∆PMM_P*ars*:M cultures at point 0 h and 72 h with the different arsenite concentrations were analyzed by western blot with specific antibodies against PGM and PMM/PGM. αGroEL was used as loading control. A 10 µg aliquot of protein from soluble extracts was loaded in each lane. (D) Cultures of the WT, ∆PGM, ∆PMM_P*ars*:M and ∆PGM∆PMM_P*ars*:M strains grown without arsenite for 6 d were used to inoculate cultures without arsenite or with 0.5, 1, 2, or 5 µM arsenite. Glycogen in the cultures was measured at the time of inoculation (0 d) or after 5 d. Data are means ±SD from three (A) or four (D) biological replicates. In (D), significant differences between glycogen content at time 0 h and 120 h with different concentrations of arsenite were determined using paired two-tailed Student’s *t*-test. *P<0.05, **P<0.01, ***P<0.001.

To confirm the essentiality of PMM/PGM, we constructed a strain in which we could regulate the levels of this protein in the cell. In a first step, we introduced an additional copy of the *slr1334* gene under the control of the P_*arsB*_ promoter (López-Maury *et al.*, 2003) in the non-essential *nrsD* locus (García-Domínguez *et al.*, 2000; Mallen-Ponce *et al.*, 2021). This construct allows tunable expression of PMM/PGM in the presence of increasing amounts of the inducer arsenite, and can be turned off by removing the inducer from the medium. In this background, the mutation of the endogenous *slr1334* copy completely segregated when the expression of the P_*arsB*_-regulated copy was induced by arsenite, generating the ∆PMM_P*ars*:M strain (Supplementary Fig. S8). Although the ∆PMM_P*ars*:M strain was routinely maintained in media supplemented with the inducer arsenite, to study the effect of the lack of PMM/PGM in *Synechocystis*, it was necessary to grow the ∆PMM_P*ars*:M strain for 6 d without the inducer before starting the experiments. This period after turning off the P_*arsB*_-regulated *slr1334* expression was required to achieve sufficiently low levels of PMM/PGM in the ∆PMM_P*ars*:M cells before proceeding to analyze its phenotype. Then, the WT and ∆PMM_P*ars*:M cells were transferred to media supplemented or not with the inducer arsenite and growth was followed (Fig. 8A, B). In the presence of 5 µM arsenite, the ∆PMM_P*ars*:M cultures showed levels of PMM/PGM and growth rates similar to those of the WT (Fig. 8A, B). However, in the absence of arsenite, PMM/PGM was undetectable by western blot and culture growth ceased (Fig. 8A–C). This confirms that PMM/PGM is an essential protein in *Synechocystis*.

To confirm that the limited carbon flux to and from glycogen in the absence of PGM was provided by PMM/PGM, we designed a new mutant. The *sll0726* gene (coding for PGM) was deleted in the ∆PMM_P*ars*:M strain, generating the ∆PGM∆PMM_P*ars*:M strain. This strain lacks PGM and has regulated expression of PMM/PGM. As in the case of ∆PMM_P*ars*:M, this strain also required a period of 6 d without the inducer arsenite to achieve sufficiently low levels of PMM/PGM before starting the experiments. In the presence of the inducer arsenite, the expression of PMM/PGM was induced in the ∆PGM∆PMM_P*ars*:M strain and the culture was viable (Supplementary Fig. S9). As expected, in the absence of the inducer, PMM/PGM was not expressed and the ∆PGM∆PMM_P*ars*:M strain was not viable (Supplementary Fig. S9). Thus this strains allowed us to turn down the levels of PMM/PGM and evaluate its contribution to glycogen synthesis in the absence of PGM. We determined the glycogen content of the ∆PGM∆PMM_P*ars*:M strain in early and mid-exponential growth phases in the presence of increasing amounts of arsenite and hence increasing amounts of PMM/PGM (Fig. 8D). In the absence of arsenite or in the presence of up to 2 µM arsenite, the amount of glycogen decreased or did not increase after 5 d. The addition of 5 µM of inducer was required to allow glycogen accumulation in the ∆PGM∆PMM_P*ars*:M strain (Fig. 8D). This strongly suggests that there is no other enzyme in addition to PGM and PMM/PGM catalyzing an efficient interconversion between G1P and G6P.

## Discussion

Carbon polymer reserves are a critical element for adequate fitness and survival after different environmental stresses in organisms from prokaryotes to mammals. Cyanobacteria, as photosynthetic organisms especially abundant in aquatic ecosystems, are particularly sensitive to specific environmental stresses, including nutritional deficit, light perturbation (light intensity and day–night regimes), metal toxicity, or salinity. In this work, we have described how a limited carbon flux to and from glycogen caused by the lack of the phosphoglucomutase (PGM) affects the performance of *Synechocystis* differently under different stresses.

*Synechocystis* has two enzymes with phosphoglucomutase activity, PGM and the bifunctional enzyme PMM/PGM. We have confirmed that PMM/PGM plays an essential role in the metabolism of *Synechocystis*, because suppression of PMM/PGM expression in the ∆PMM_P*ars*:M or ∆PGM∆PMM_P*ars*:M strains is lethal (Fig. 8A, B; Supplementary Fig. S9). *Synechocystis* PMM/PGM has been described to have both phosphoglucomutase and phosphomannomutase activity *in vitro* (Liu *et al.*, 2013), a characteristic common to the enzymes of this subgroup of the phosphohexomutase superfamily (Stiers *et al.*, 2017). The interconversion between mannose-1-phosphate and mannose-6-phosphate is required for the synthesis of GDP-mannose and lipopolysaccharides (Mills *et al.*, 2020). Furthermore, this enzyme has recently been described to carry out the synthesis of G1,6BP, a metabolite necessary for the activation of PGM and probably other members of the phosphohexomutase superfamily (Neumann *et al.*, 2022). *Synechocystis* also presents a predicted protein of the PNGM subgroup, although it has not yet been characterized. PNGMs are required for the synthesis of UDP-*N*-acetylglucosamine and have been described as essential in bacteria such as *Escherichia coli*, *Pseudomonas aeruginosa*, or *Helicobacter. pylori* (Stiers *et al.*, 2017). Thus, in the absence of G1,6BP, PNGM could remain inactive, which could, in turn, affect the viability of the cells. In addition, G1,6BP could have other as yet unidentified roles in *Synechocystis* metabolism, as has been described for the regulation of other key enzymes in mammals (Carreras *et al.*, 1986). The introduction of enzymes from other organisms with strictly phosphomannomutase or glucose-1,6-bisphosphate synthase activity in the ∆PGM∆PMM_P*ars*:M strain could shed some light on the weight of each of the activities of PMM/PGM in its essentiality. In any case, the essential function of PMM/PGM would probably be in a process independent of glycogen metabolism, since glycogen-less mutants are perfectly viable. Further work is required to decipher the role of PMM/PGM in *Synechocystis*.

On the other hand, enzymes belonging to the PGM subgroup have been described to play an important role not only in the metabolism of storage polysaccharide, but also in aspects such as bacterial capsular polysaccharide and cell wall biosynthesis (Patel *et al.*, 2019; McFarland *et al.*, 2021), pathogenesis (Ugalde *et al.*, 2000), protein glycosylation (Tegtmeyer *et al.*, 2014), or development of gametophytes in plants (Egli *et al.*, 2010). The identification of the regulation of PGM activity by phosphorylation in response to environmental factors, such as nutrient deprivation (Doello *et al.*, 2022), emphasized its role as a key element in metabolism. This is particularly evident as mutants carrying a non-phosphorylatable version of PGM are not able to survive the dormant state induced by nitrogen deprivation (Doello *et al.*, 2022).

In this work, we have focused on the role of PGM in the connection between central carbon metabolism and glycogen. We have always detected glycogen in our mutant lacking PGM in *Synechocystis*, with levels ranging between ~50% and 15% with respect to the WT depending on the culture conditions (Figs 2–6; Supplementary Fig. S2). There are discrepancies in this aspect in previous work on *Synechocystis*. While Liu *et al.* (2013) also detected glycogen accumulation in a *Synechocystis* mutant lacking PGM, Doello *et al.* (2022) reported no glycogen in their mutant. These discrepancies in glycogen content may not be attributed to different culture conditions, as we have tried a wide variety of growth conditions and glycogen was detected in all of them (Figs 2–6; Supplementary Fig. S2). However, we cannot rule out an effect of the genetic background, as several WT backgrounds are used across different laboratories, or even the presence of spontaneous mutations acquired during the segregation process.

Reports in the literature on the storage polysaccharide content of mutants lacking PGM in other organisms range from a 50% reduction in glycogen levels in the double *pgm1/2* mutant in yeast (Boles *et al.*, 1994; Daran *et al.*, 1997) to near starchless in the plastidial PGM (pPGM) mutant in Arabidopsis (Bahaji *et al.*, 2014) or complete glycogen less in *E. coli* (Eydallin *et al.*, 2007). In the case of Arabidopsis, the residual starch content in the absence of pPGM has been proposed to be due to the existence of an alternative source of ADP-Glc that would not require the action of pPGM or AGP (Bahaji *et al.*, 2014). This would not be the case in *Synechocystis*, as mutants lacking AGP are completely devoid of glycogen. In contrast, it is more plausible that, due to its phosphoglucomutase activity, PMM/PGM would be the main candidate for G1P synthesis in glycogen metabolism in the absence of PGM. As PMM/PGM is essential, it is not easy to ascribe glycogen synthesis detected in the ∆PGM strain to PMM/PGM, because suppression of PMM/PGM expression affects cell viability. However, our data show that down-regulation of PMM/PGM to non-lethal levels causes decreased glycogen levels, indicating that PMM/PGM is involved in glycogen synthesis at least in the absence of PGM. Phosphoglucomutase activity has been described in enzymes belonging to other subgroups of the phosphohexomutase superfamily due to some degree of substrate promiscuity (Mitra *et al.*, 2010; Bandini *et al.*, 2012; Patel *et al.*, 2019). Although *Synechocystis* has an enzyme assigned to the PNGM subgroup, our data strongly suggest a negligible effect, if any, of any other enzyme in addition to PGM and PMM/PGM in the interconversion of G1P and G6P in glycogen metabolism (Fig. 8D). Furthermore, overexpression of PMM/PGM in a strain lacking PGM increases the glycogen content and recovers the response to different types of stresses (Fig. 7; Supplementary Fig. S6) which reinforces the secondary role of PMM/PGM in glycogen metabolism.

Due to the presence of PMM/PGM in *Synechocystis*, in the ∆PGM strain the carbon flux between central carbon metabolism and carbon storage is not completely blocked, as in extensively characterized mutants lacking *agp* or *glgA* genes, but is constricted by the limited capacity of PMM/PGM. This provides additional information on the degree of dependence on glycogen metabolism for an adequate response to environmental stresses. The stresses examined can be classified as low/moderate and highly dependent on glycogen metabolism. The low/moderate dependency category includes salt stress and long-day conditions. The synthesis of the osmolytes GG and sucrose to face salt stress is based on G1P (Fig. 1). This metabolite can be generated from photosynthetic fixed carbon through PGM (and/or PMM/PGM) or by glycogen mobilization by glycogen phosphorylases. In this regard, it is also interesting to note that the addition of NaCl to the WT or ∆PGM strains induced the mobilization of glycogen, which was greater in the WT than in the ∆PGM strain (Fig. 6D). Consistent with previous results in *Synechococcus elongatus* PCC 7942 (Qiao *et al.*, 2018), this suggests that at least part of the G1P required for osmolyte synthesis could be derived from glycogen mobilization to G1P by glycogen phosphorylases. This would also explain the slower kinetics of GG accumulation in the ∆PGM strain (with reduced glycogen levels) (Fig. 6B) and, mainly, in sucrose accumulation in the glycogen-less ∆AGP strain (Fig. 6C), although G1P levels seem higher in the ∆PGM than in the WT strain (Fig. 7G).

Glycogen does not appear to play a critical role in survival of *Synechocystis* cells in long-day light–dark regimes (Fig. 3A). However, exposure to cycles with extended dark periods (16 h dark/8 h light) had a negative impact on the ∆AGP and ∆PGM strains (Fig. 3B). In this case, the existence of a flux, although limited, between glycogen and central carbon metabolism in the ∆PGM strain represents a clear advantage over the glycogen-less ∆AGP strain, which is not viable under these conditions (Fig. 3B). Glycogen would act here as a source of carbon and reducing power through its respiration in the dark (Preiss, 1984; Gründel *et al.*, 2012) and to replenish the intermediates of the Calvin–Benson cycle depleted during the dark period (Makowka *et al.*, 2020; Shinde *et al.*, 2020).

On the other hand, both exposure to high light intensity and response to nitrogen deficiency require a fully functional glycogen metabolism. In these situations, the phosphoglucomutase activity of the endogenous PMM/PGM is clearly insufficient and the ∆PGM strain has a phenotype like the ∆AGP strain (Figs 2, 4). In the case of the response to nitrogen deficiency, one of the main characteristics of adaptation to this nutritional condition is a fast and prominent accumulation of glycogen that reaches its maximum ~48 h after nitrogen removal under our conditions (Fig. 4C). During this period, carbon is relocated from the recycling of phycobiliproteins, which will be partially accumulated as glycogen (Forchhammer and Schwarz, 2019). This process involves an intense flux of carbon into glycogen that requires high levels of interconversion between G6P and G1P provided by PGM that cannot be supplied by endogenous PMM/PGM and results in an accumulation of G6P in the ∆PGM strain (Fig. 7G). Nitrogen starvation also induces in the ∆PGM strain a metabolic overflow and a blockage of the bleaching process as occurs with the ∆AGP strain (Fig. 4; Supplementary Fig. S3), even when the ∆PGM strain accumulates reduced glycogen levels (Fig. 4C). In any case, these reduced glycogen levels represent an advantage for the ∆PGM strain, which survives longer periods under nitrogen deficiency than the ∆AGP strain (Supplementary Fig. S4). It is interesting to note that after nitrogen replenishment, the ∆PGM strain can resume vegetative growth at rates comparable with those of the WT (Fig. 5B) despite the reduced glycogen levels (Fig. 5C). This could be due to a lower energy requirement for the awakening program, as the phycobilisomes were still present (Fig. 5A; Supplementary Fig. S3A).

Finally, one of the classic strategies in metabolic modification of cyanobacteria for biotechnological purposes has been to eliminate carbon reserves, mainly glycogen, to redirect the fixed carbon toward the production of compounds of interest. This strategy has generally been carried out by deleting *agp* or *glgA* genes. However, these modifications generally lead to a reduction in fitness and sensitivity to various stresses (reviewed in Luan *et al.*, 2019), including those analyzed in this work, which ultimately reduce the productivity of the modified strains. Reducing, but not completely abolishing, glycogen storage by limiting phosphoglucomutase activity, as described here, is an alternative that improves stress resistance and deserves further exploration.

## Supplementary data

The following supplementary data are available at *JXB* online.

Table S1. Oligonucleotides used in this work.

Fig. S1. Generation and segregation of the ∆PGM and ∆PMM* mutant strains.

Fig. S2. Glycogen accumulation in the ∆PGM strain cultivated in media with different availabilities of HCO_3_.

Fig. S3. Degradation of the phycobilisomes and *F*_o_ and *F*_m_ photosynthetic parameters of the ∆PGM strain during nitrogen deprivation.

Fig. S4. Appearance and recovery of the WT, ∆AGP, and ∆PGM strains after 15 d of nitrogen deprivation.

Fig. S5. Phosphoglucomutase activity of WT, OE:M, ∆PGM, and ∆PGM_OE:M after 24 h exposure to high light, nitrogen deprivation, or salt stress, and levels of proteins involved in glycogen synthesis.

Fig. S6. Phenotype of PMM/PGM-overexpressing strains grown under diel regimes.

Fig. S7. Phenotype of OE:M under high light.

Fig. S8. Generation of the ∆PMM_P*ars*:M strain.

Fig. S9. Growth and PMM/PGM levels of the ∆PGM∆PMM_P*ars*:M strain cultivated with different amounts of arsenite.

erac474_suppl_Supplementary_Table_S1_Figures_S1-S9

## Data Availability

All data supporting the findings of this study are available within the paper and within its supplementary data published online.
