# Supplementary material for: Stress response requires an efficient connection between glycogen and central carbon metabolism by phosphoglucomutases in cyanobacteria
Source: J Exp Bot. 2022 Dec 1;74(5):1532–50. doi: 10.1093/jxb/erac474 (PMC10010611; doi:10.1093/jxb/erac474)
Supplement: erac474_suppl_Supplementary_Table_S1_Figures_S1-S9 [file erac474_suppl_supplementary_table_s1_figures_s1-s9.pdf]

SUPPLEMENTARY MATERIAL

Article title:

Stress response requires efficient glycogen and central carbon metabolism connection by phosphoglucomutases in cyanobacteria

Authors and institutions:

Pablo Ortega-Martínez[1,2], Miguel Roldán[2], Sandra Díaz-Troya[1,2] and Francisco J. Florencio[1,2].

1. Instituto de Bioquímica Vegetal y Fotosíntesis, Universidad de Sevilla, Consejo Superior de Investigaciones Científicas, Américo Vespucio 49, Sevilla, 41092, Spain

2. Departamento de Bioquímica Vegetal y Biología Molecular, Facultad de Biología, Universidad de Sevilla, Profesor García González s/n, Sevilla, 41012, Spain.

Table S1. Oligonucleotides used in this work

Primer name	Sequence	Use
OL68	GGTCATTAACTGTGCCTTCGTCAC	Analysis of ∆PGM segregation and construction of plasmid pGT∆sll0726::Km
OL69	GGTCGAGGATCCCCAAATCCTAGCCAAAAAGG	Construction of plasmid pGT∆sll0726::Km
OL70	ATTTGGGGATCCTCGACCCGGTCAATGTTCC	
OL71	GCTAATTGGGCTGGGGGCCAATTAG	
OL72	GATAGGAAGAACTCCCAACTCTACTG	Analysis of ∆PMM* and ∆PMM_P*ars*:M segregation and construction of plasmid pGT∆slr1334::Ery
OL73b	TAAATCGGATCCGACAGACCTAGTTTTTGTGGAT	Construction of plasmid pGT∆slr1334::Ery
OL74b	TCTGTCGGATCCGATTTATTGACCACCGACGGC	
OL75	ATGGGGCGAAAATTATCTGCGTCAG	
OL78	CCGGCATATGACAAGCAGAATTAATCCC	Construction of plasmid pET28sll0726
OL79	GCCGAATTCTTAGCCCAAAGCCGAGGTAAC	Analysis of ∆PGM segregation and construction of plasmid pET28sll0726
OL80	CCGGCATATGGTTTACACTCCTGCTCC	Analysis of ∆PMM* segregation and construction of plasmid pET28slr1334
OL81	GCCGAATTCTCAGTGAGATGATTGTGCAG	Construction of plasmid pET28slr1334
OL184	ATCGGCAAATACCCGG	Analysis of ∆PMM* and ∆PMM_P*ars*:M segregation
OLEryR	AAAAGCTTAGGATCGATCCTCTAGCTAGA	Analysis of ∆PMM* and ∆PMM_P*ars*:M segregation
OLg204	AAGCGCGCCTTTCACTGCTTGCGGAACC	Analysis of ∆PMM_P*ars*:M segregation
OLKamR	AACGATCCTTCATCCTGTCTC	Analysis of ∆PGM segregation

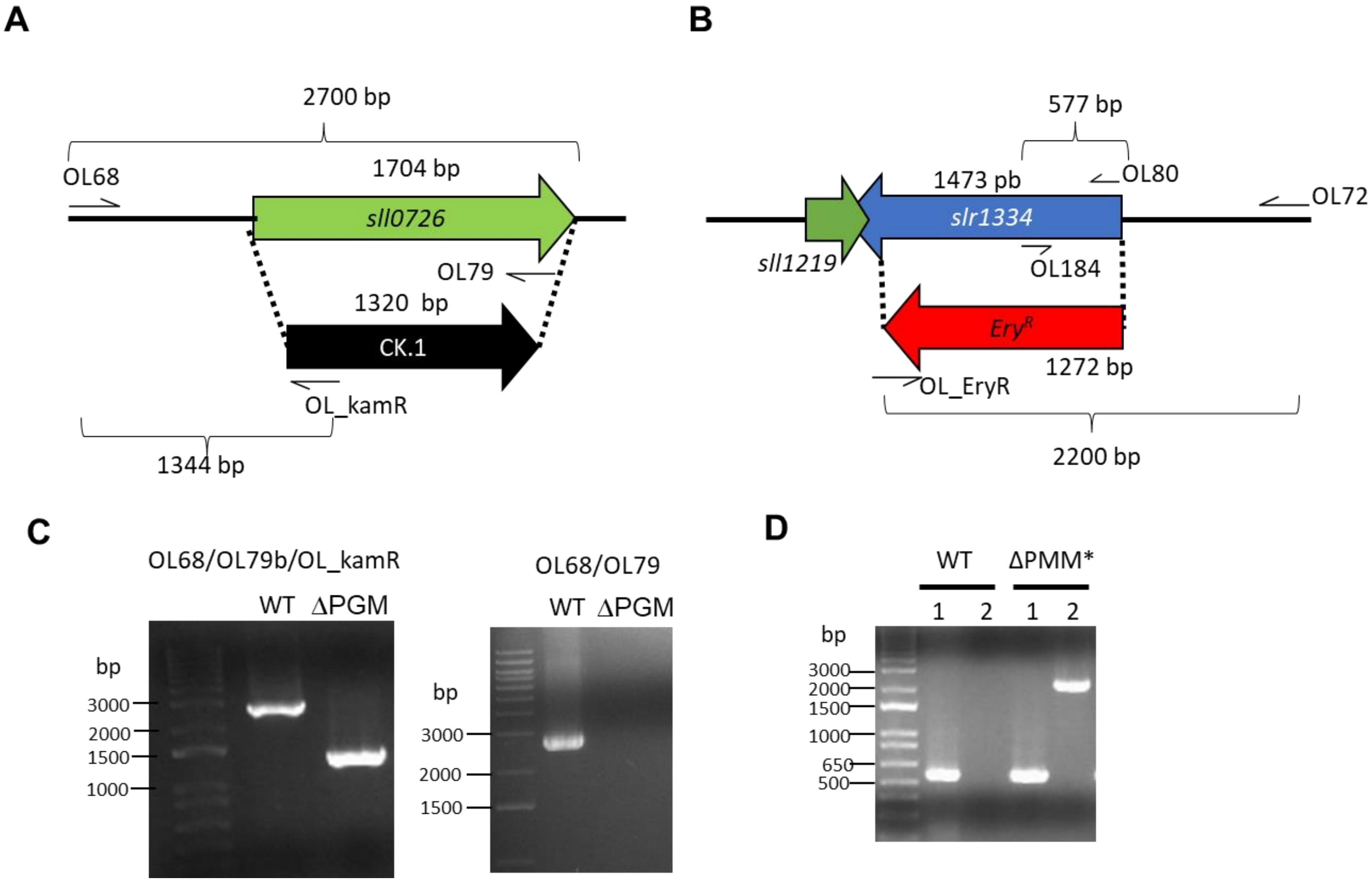

Fig. S1. Generation and segregation of the ∆PGM and ∆PMM* mutant strains. (A) Scheme of the strategy for the deletion of *sll0746* (coding for PGM) to generate the ∆PGM strain. (B) Scheme of the strategy for the deletion of *slr1334* (coding for PMM/PGM) to generate the ∆PMM* strain. (C) PCR with primers depicted in A to analyze the grade of segregation of the ∆PGM strain. (D) PCR with primers depicted in B to analyze the grade of segregation of the ∆PMM* strain. Primers in line 1: OL80/OL184; primers in line 2: OL72/OL_EryR. CK.1 and *Ery*^{R} indicate kanamycin and erythromycin resistance cassettes, respectively.

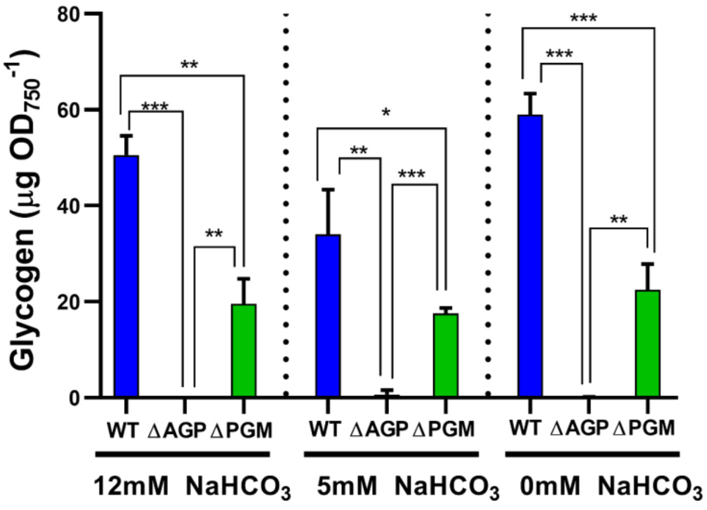

Fig. S2. Glycogen accumulation in the ∆PGM strain cultivated in flasks with media with different availabilities of $NaHCO_3$. WT, ∆AGP and ∆PGM cells were inoculated at an $OD_{750\ nm}$ of 0.5 in Erlenmeyer flasks with BG11 containing 12 mM, 5 mM o no $NaHCO_3$ and cultivated in continuous shaking. Glycogen content was measured after 7 days. Data are means ±SD from three biological replicates. Significant differences were determined using unpaired two-tailed Student's *t*-test: ∗*P*≤0.05, ∗∗*P*≤0.01, ∗∗∗*P*≤0.001.

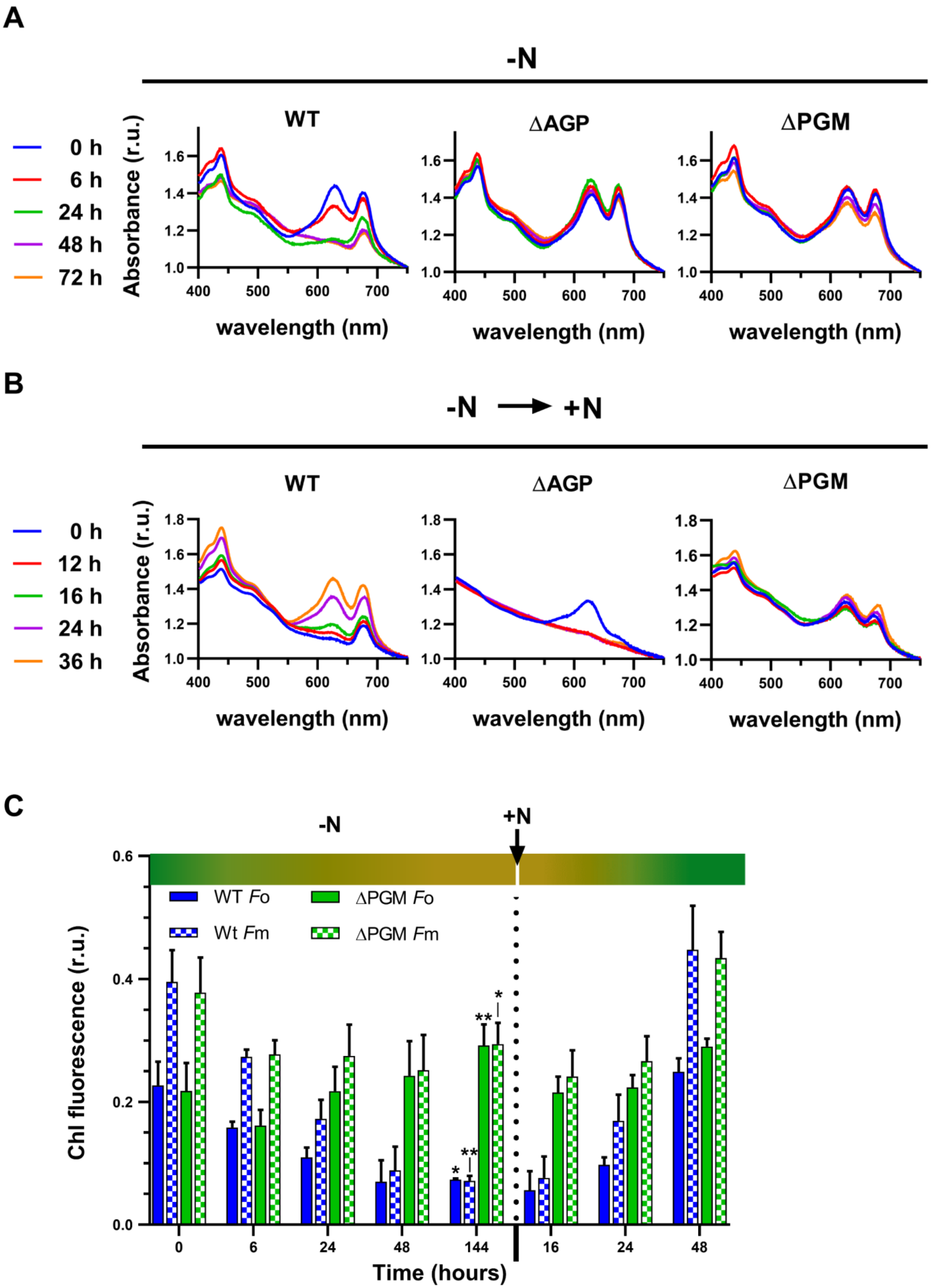
A
-N
WT
ΔAGP
ΔPGM
0 h
6 h
24 h
48 h
72 h
Absorbance (r.u.)
1.0
1.2
1.4
1.6
400
500
600
700
wavelength (nm)
B
-N → +N
WT
ΔAGP
ΔPGM
0 h
12 h
16 h
24 h
36 h
Absorbance (r.u.)
1.8
wavelength (nm)
C
-N
+N
WT Fo
Wt Fm
ΔPGM Fo
ΔPGM Fm
Chl fluorescence (r.u.)
0.0
0.2
0.4
0.6
*
**
0
6
24
48
144
16
24
48
Time (hours)

Fig. S3. Degradation of the phycobilisomes and *F*o and *F*m photosynthetic parameters of the ΔPGM strain during nitrogen deprivation. Cells of the WT, ΔAGP, and ΔPGM strains were grown in nitrogen-repleted medium to mid-exponential growth phase, washed twice in nitrogen-free medium and transferred to nitrogen-free medium. After 6 days of nitrogen deprivation, cells were transferred again to nitrogen-repleted medium. (A) Whole cell spectra of WT, ΔAGP and ΔPGM cultures over three days of nitrogen deprivation. (B) After 6 days of nitrogen deprivation of the WT, ΔAGP and ΔPGM cultures, nitrogen was replenished. Whole cell spectra of the cultures during over 36 hours after nitrogen replenishment. In (A) and (B) spectra were normalized to absorbance at 750 nm. (C) Maximum fluorescence yield (*F*m) and minimal fluorescence (*F*o) of WT and ΔPGM cultures during nitrogen deprivation and after nitrogen replenishment measured by PAM florometry. *F*m was determined in the presence of DCMU (20 µM) under growth light (50 µE m^{-2} s^{-1}). Green to orange and orange to green bars represent cultivation in media without or with nitrogen, respectively. Data in (C) are means ±SD from three biological replicates. Significant differences in *F*o and *F*m values of the WT and ΔPGM strains at time 144 hours after nitrogen removal compared with their respective values at time 0 hours were determined using paired two-tailed Student's *t*-test. $*P<0.05$, $**P<0.01$, $***P<0.001$.

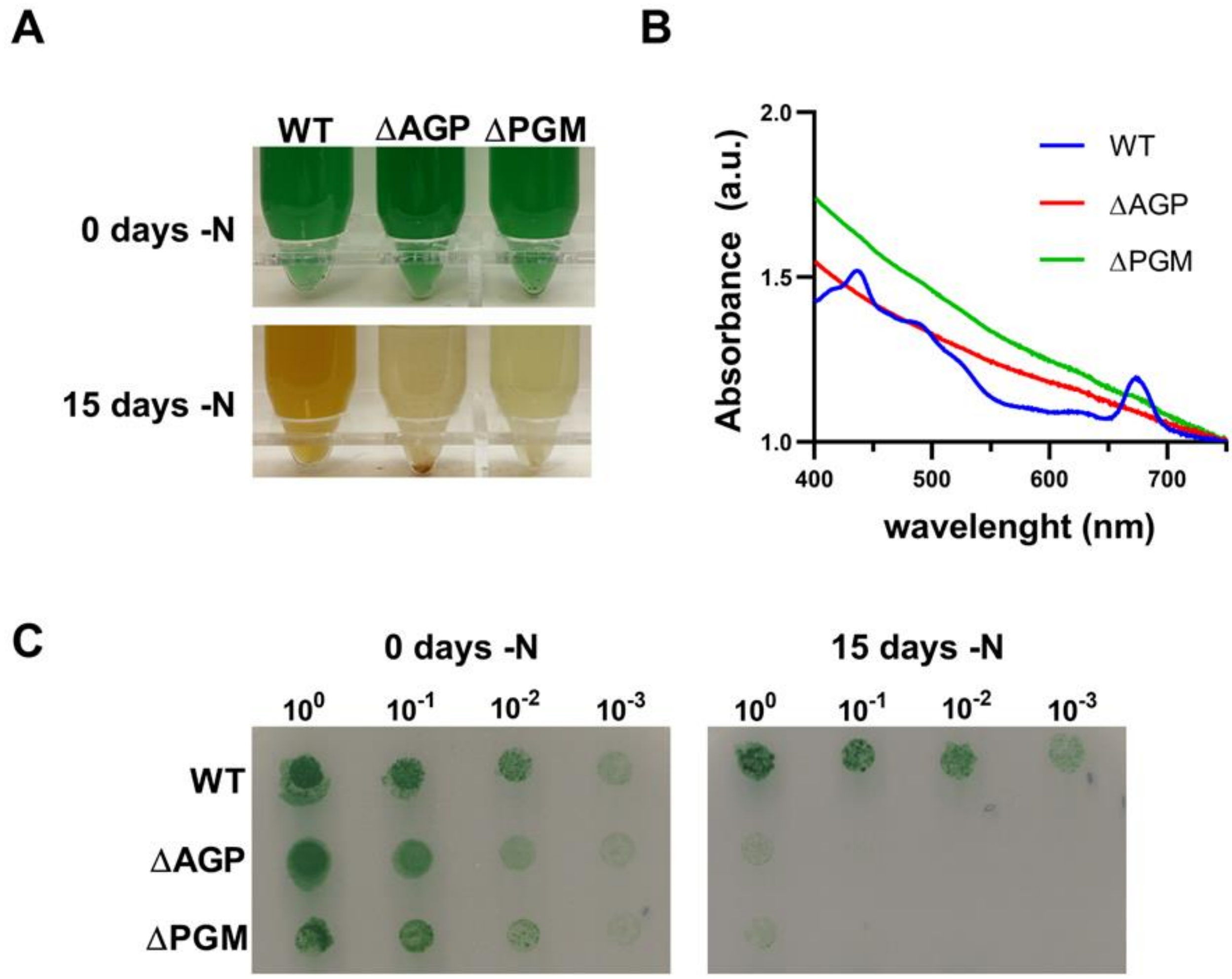

Fig. S4. Appearance and recovery of the WT, ΔAGP and ΔPGM strains after 15 days of nitrogen deprivation. WT, ΔAGP, and ΔPGM cultures were grown to mid-exponential phase in BG11C, washed in nitrogen-free medium ($BG11_0C$) and transferred to $BG11_0C$ for 15 days. (A) Photographs of the WT, ΔAGP, and ΔPGM cultures immediately after and 15 days after nitrogen removal. (B) Whole cell spectra normalized to absorbance at 750 nm of WT, ΔAGP, and ΔPGM cultures after cultivation in $BG11_0C$ medium for 15 days. (C) Viability assay of the WT, ΔAGP, and ΔPGM strains. Aliquots of the WT, ΔAGP, and ΔPGM cultures immediately after and 15 days after nitrogen removal were spotted on BG11C plates and cultivated under continuous light for 6 days. Dilutions are indicated above each panel.

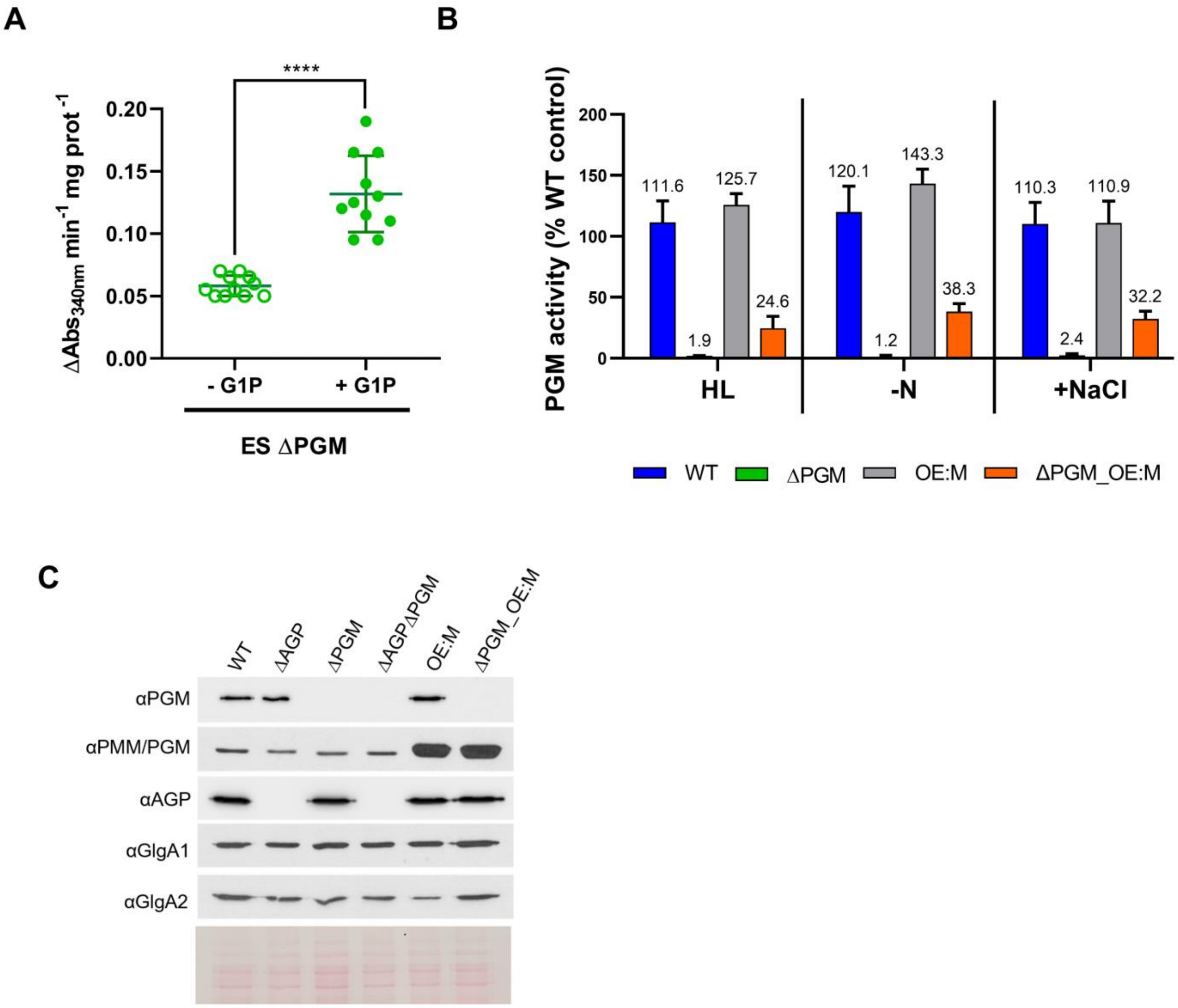
A
****
ΔAbs340nm min-1 mg prot -1
0.20
0.15
0.10
0.05
0.00
- G1P
+ G1P
ES ΔPGM
B
PGM activity (% WT control)
200
150
100
50
0
111.6
1.9
125.7
24.6
120.1
1.2
143.3
38.3
110.3
2.4
110.9
32.2
HL
-N
+NaCl
WT
ΔPGM
OE:M
ΔPGM_OE:M
C
WT
ΔAGP
ΔPGM
ΔAGPΔPGM
OE:M
ΔPGM_OE:M
αPGM
αPMM/PGM
αAGP
αGlgA1
αGlgA2

Fig. S5. Phosphoglucomutase activity of WT, OE:M, ∆PGM and ∆PGM_OE:M after 24 hours exposition to high light, nitrogen deprivation or salt stress and levels of proteins involved in glycogen synthesis. (A) Confirmation of the significant difference between low phosphoglucomutase activity of ∆PGM strain and background noise. Raw data from phosphoglucomutase activity assays with cell soluble extract (ES) from ∆PGM strain with and without the addition of the substrate G1P. (B) WT, OE:M, ∆PGM and ∆PGM_OE:M T and OE:M cells grown under 50 µE m-2 s-1 were exposed to high light (200 µE m-2 s-1), transferred to nitrogen depleted media, or supplemented with 500 mM NaCl. After 24 hours, samples were collected and phosphoglucomutase activity was measured in cells extracts as indicated in Material and Methods. Values were referenced to WT phosphoglucomutase activity under control conditions (Fig. 7B). (C) Ten µg of soluble extract proteins from WT, ∆AGP, ∆PGM, ∆AGP∆PGM, OE:M, and ∆PGM_OE:M grown under standard conditions were resolved by SDS-PAGE and probed with specific antibodies against PGM, PMM/PGM, AGP, GlgA1, and GlgA2. Ponceau staining of the membrane is included as loading control. Data in (A) and (B) are means ±SD from three biological replicates. In (B), no significant differences compared with their corresponding strain under control conditions (Fig. 7B) were found using unpaired two-tailed Student’s t-test. In (C), significant difference was determined using paired two-tailed Student’s t-test. ∗P<0.05, ∗∗P<0.01, ∗∗∗P<0.001, ∗∗∗∗P<0.0001.

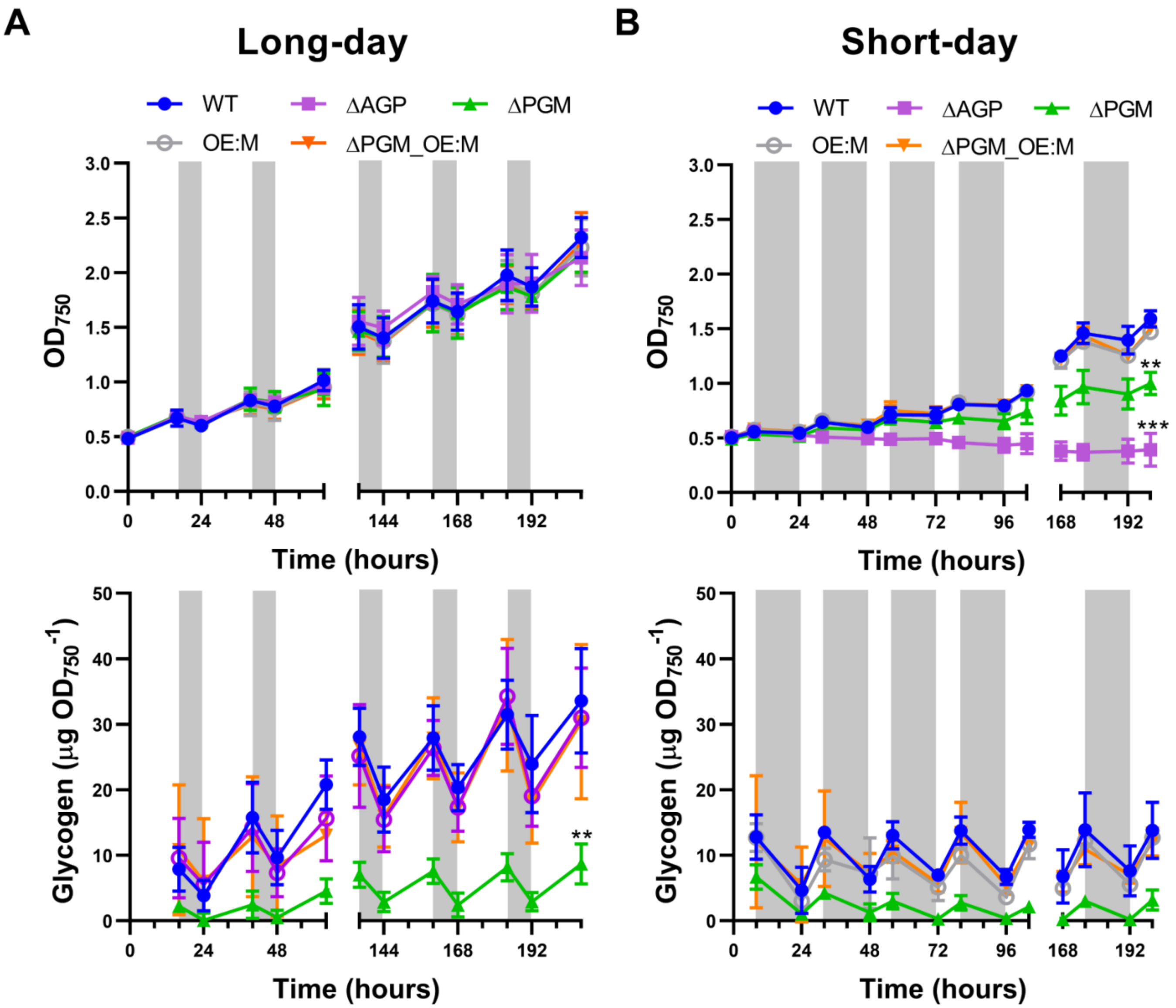

Fig. S6. Phenotype of PMM/PGM overexpressing strains grown under diel regimes. WT, ΔAGP, ΔPGM, OE:M and ΔPGM_OE:M cells were cultivated under (A) long-day (16 hours light/8 hours dark) or (B) short-day (8 hours light/16 hours dark) conditions. White and gray areas represent light and dark periods, respectively. Growth and glycogen content were determined. Data are means ±SD from three biological replicates. Significant differences in $OD_{750\ nm}$ or glycogen content at the end of the experiment compared with the WT strain were determined using paired two-tailed Student's *t*-test: $*P<0.05$, $**P<0.01$, $***P<0.001$. Color of "*" matches color of its corresponding strain.

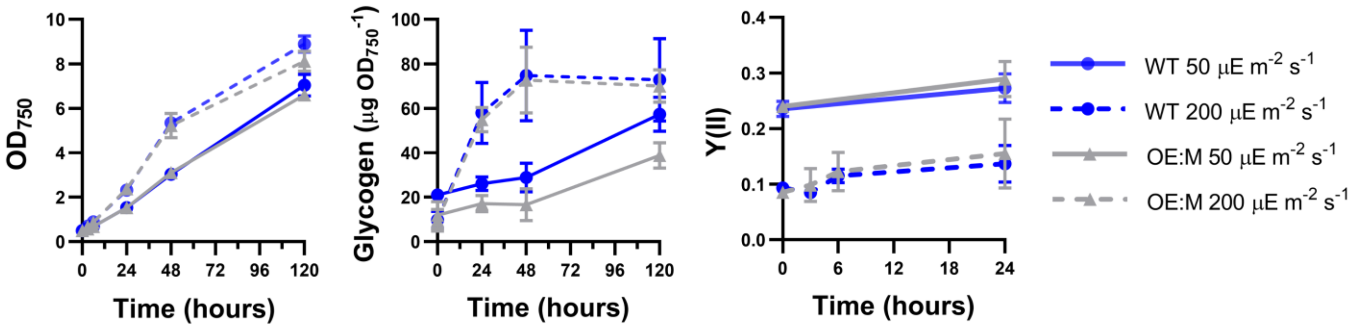

Fig. S7. Phenotype of OE:M under high light. WT and OE:M cells grown under 50 µE m^{-2} s^{-1} were used to inoculate cultures at 50 or 200 µE m^{-2} s^{-1}. Growth curves, glycogen content, and PSII quantum yields (YII) measured by PAM fluorometry of WT and OE:M strains cultivated under 50 or 200 µE m^{-2} s^{-1}. Data are means ±SD from three biological replicates.

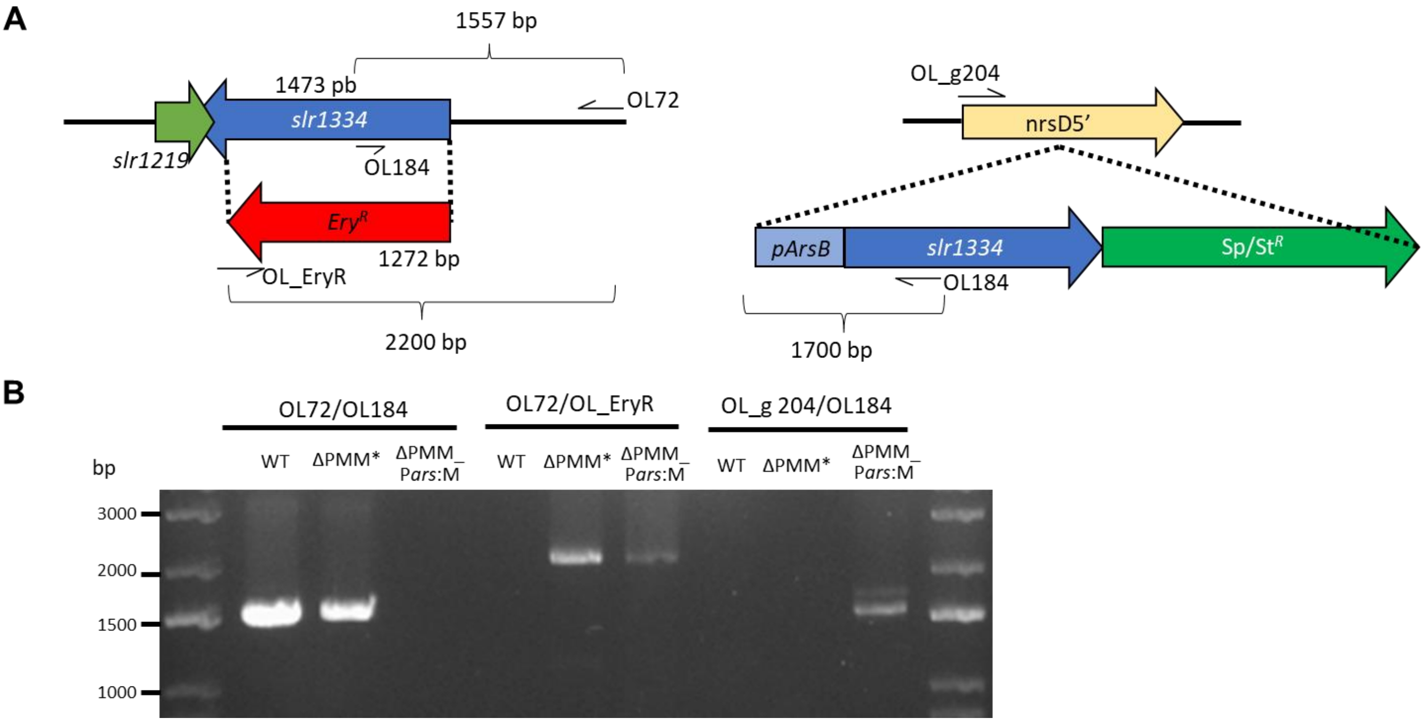

Fig. S8. Generation of the ∆PMM_P*ars*:M strain. (A) Scheme of the strategy for the deletion of *slr1334* gene and insertion of the regulated copy of *slr1334* in the *nrsD* locus. Primers used to analyze the segregation of mutants are included. (B) PCR with the primers shown in A to analyze the grade of segregation of the generated mutant strain.

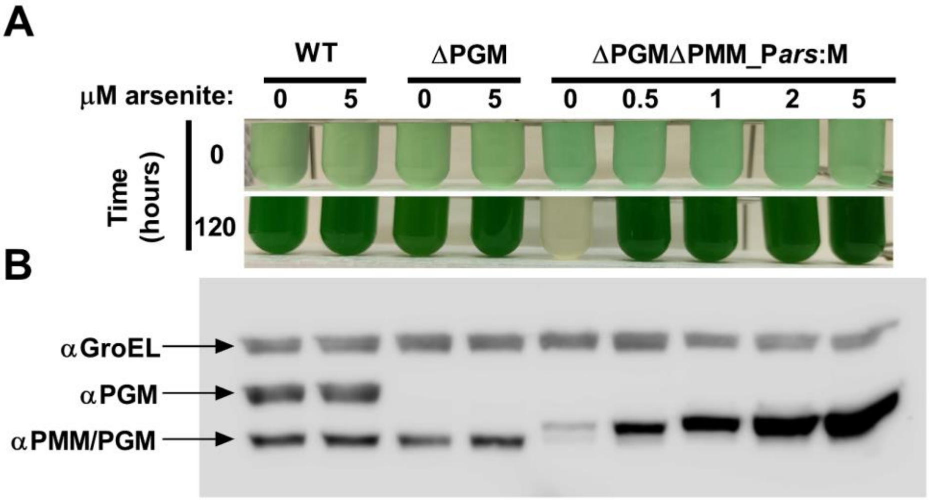

Fig. S9. Growth and PMM/PGM levels of the ΔPGMΔPMM_P*ars*:M strain cultivated with different amounts of arsenite. WT, ΔPGM, and ΔPGMΔPMM_P*ars*:M cells were cultured in the absence of arsenite for six days and then transferred to medium with the indicated amounts of arsenite (0, 0.5, 1, 2 or 5 µM arsenite). Photographs of the cultures before and five days after arsenite addition (upper panel). PGM and PMM/PGM levels in WT, ΔPGM and ΔPGMΔPMM_P*ars*:M cells three days after arsenite addition (lower panel). Ten µg of soluble proteins were resolved by SDS-PAGE and probed with specific antibodies against PGM and PMM/PGM. Levels of GroEL was used as a control of equal loading.
